# Trends of respiratory viruses and factors associated with severe acute respiratory infection in patients presenting at a university hospital: a 6-year retrospective study across the COVID-19 pandemic

**DOI:** 10.3389/fpubh.2025.1494463

**Published:** 2025-03-28

**Authors:** Judith Carolina De Arcos-Jiménez, Pedro Martinez-Ayala, Ernestina Quintero-Salgado, Rosendo Lopez-Romo, Jaime Briseno-Ramirez

**Affiliations:** ^1^Laboratory of Microbiological, Molecular, and Biochemical Diagnostics (LaDiMMB), CUTlajomulco, University of Guadalajara, Tlajomulco de Zuñiga, Jalisco, Mexico; ^2^State Public Health Laboratory, Guadalajara, Mexico; ^3^Hospital Civil de Guadalajara “Fray Antonio Alcalde”, Guadalajara, Mexico; ^4^Division of Health, CUTlajomulco, University of Guadalajara, Tlajomulco de Zuñiga, Jalisco, Mexico

**Keywords:** COVID-19, respiratory viruses, influenza, severe acute respiratory infection (SARI), respiratory virus trends, respiratory syncytial virus (RSV), epidemiology

## Abstract

**Background:**

The COVID-19 pandemic significantly disrupted the epidemiology of respiratory viruses, altering seasonal patterns and reducing circulation. While recovery trends have been observed, factors associated with severe acute respiratory infections (SARIs) during pre- and post-pandemic periods remain underexplored in middle-income countries.

**Objective:**

This study aimed to analyze the trends in respiratory virus circulation and identify factors associated with SARI in patients attending a tertiary care university hospital in western Mexico over a six-year period spanning the pre-pandemic, pandemic, and post-pandemic phases.

**Methods:**

A retrospective study was conducted using data from 19,088 symptomatic patients tested for respiratory viruses between 2018 and 2024. Viral trends were analyzed through interrupted time series (ITS) modeling, incorporating locally estimated scatterplot smoothing (LOESS) and raw positivity rates. Additionally, ITS analysis was performed to evaluate temporal changes in SARI proportions across different phases of the pandemic. Multivariate logistic regression models were applied to determine independent risk factors for SARI across different time periods.

**Results:**

During the pandemic (2020–2021), respiratory virus positivity rates significantly declined, particularly for influenza, which experienced a sharp reduction but rebounded post-2022. Respiratory syncytial virus (RSV) demonstrated a delayed resurgence, whereas other respiratory viruses exhibited heterogeneous rebound patterns. ITS modeling of SARI proportions revealed a significant pre-pandemic increasing trend, followed by a slower rise during the pandemic, and a sharp post-pandemic drop in early 2022, before resuming an upward trajectory. Among older adults (>65 years), a marked increase in SARI was observed at the beginning of the pandemic, while younger groups showed more stable patterns. Logistic regression identified advanced age, male sex, cardiovascular disease, obesity, and immunosuppression as major risk factors for SARI, while vaccination consistently showed a protective effect across all periods and subgroups.

**Conclusion:**

The COVID-19 pandemic induced persistent shifts in respiratory virus circulation, disrupting seasonal dynamics and modifying the burden of SARI. The findings underscore the importance of continuous surveillance, targeted vaccination programs, and early diagnostics to mitigate severe outcomes. These results highlight the need for adaptive public health strategies in middle-income countries to address evolving respiratory disease threats.

## 1 Introduction

The dynamics of respiratory viruses, including influenza, respiratory syncytial virus (RSV), parainfluenza virus (HPIV), and metapneumovirus (hMPV), have exhibited substantial variations across the pre-pandemic, pandemic, and post-pandemic periods of COVID-19 (1). Before the COVID-19 pandemic, respiratory viruses followed well-defined seasonal patterns, and influenza was the primary public health concern among respiratory infections (2). The COVID-19 pandemic profoundly disrupted the epidemiology of respiratory viruses (3). Public health measures implemented to curb the spread of severe acute respiratory syndrome coronavirus 2 (SARS-CoV-2), including mask-wearing, social distancing, and travel restrictions, drastically reduced the circulation of other respiratory viruses (3). For instance, positivity rates for influenza A, influenza B, and RSV dropped to nearly undetectable levels in many regions during the first year of the pandemic, as demonstrated by both hospital-based studies and population-level data analyses (2–4). With the relaxation of COVID-19 control measures, some respiratory viruses have resurged, although not all have returned to their pre-pandemic patterns (5–7). The detection of influenza and RSV has begun to rise again; however, their seasonality and epidemic magnitude initially appear to have shifted (7, 8). Additionally, the frequency of severe acute respiratory infections (SARI) and its role as a cause of hospitalization may have also been affected (5, 6, 8).

The 2009 influenza A H1N1 pandemic influenced the seasonality and age distribution of other respiratory viruses, highlighting the potential for pandemics to induce lasting shifts in respiratory virus epidemiology (9). Changes in the frequency and prevalence of respiratory viruses during and after the COVID-19 pandemic can be attributed to a combination of factors, primarily due to public health measures and shifts in human behavior (10–12). Interventions such as mask-wearing and social distancing played a critical role in significantly reducing the transmission of respiratory viruses (10, 13, 14). Although the presence of a dominant virus like SARS-CoV-2 may have influenced the spread of other viruses through competition for ecological niches, these patterns were likely driven by changes in human behavior, such as improved personal hygiene and altered healthcare-seeking practices (12, 15–18).

Viral interference between SARS-CoV-2 and other respiratory viruses has been a subject of significant interest (11). Specifically, interactions between SARS-CoV-2 and viruses such as influenza A and RSV are influenced by multiple immune and molecular mechanisms (19). Influenza A and RSV can elicit a strong interferon (IFN) response that inhibits SARS-CoV-2 replication, particularly when these infections occur prior to or concurrently with SARS-CoV-2 (11, 19). Conversely, SARS-CoV-2 induces a weaker IFN response, limiting its ability to interfere with influenza virus replication (11, 19, 20). Additionally, the SARS-CoV-2 ORF3a protein modulates ion channels and triggers pro-inflammatory responses, potentially altering viral replication dynamics (21). The nucleocapsid protein enhances viral RNA transcription and may influence the cellular environment, affecting co-infecting viruses (22). Furthermore, SARS-CoV-2’s broad receptor usage, including ACE2 and neuropilin-1, may create competition for cellular entry, impacting co-infections (23). Its dysregulated inflammatory response, characterized by cytokine storms and autoimmune activation, can further suppress or exacerbate other viral infections (24, 25). These interactions highlight the complex interplay between SARS-CoV-2 and other respiratory viruses during the COVID-19 pandemic.

SARI, caused by various respiratory viruses, is a leading cause of hospitalizations, posing a significant health concern, especially among vulnerable populations, and placing substantial strain on healthcare systems during peak seasons or outbreaks (8, 26, 27). These vulnerable groups include pediatric patients under 2 years of age, older individuals, immunocompromised patients, and individuals with underlying health conditions (28–30). The presence of comorbidities such as cardiovascular disease, chronic pulmonary disease, chronic neurologic disease, and asthma significantly increases the risk of severe respiratory infections and poor outcomes (31–34). Immunocompromised patients are especially susceptible to severe respiratory infections, and respiratory viruses are known to exacerbate chronic respiratory conditions (34, 35). These infections can result in severe outcomes such as acute respiratory distress syndrome (ARDS) and pneumonia and are frequently complicated by bacterial coinfections (36, 37). Respiratory viruses commonly associated with severe pneumonia—whether community-acquired, hospital-acquired, or ventilator-associated—include influenza virus, human rhinovirus, parainfluenza, adenovirus, human metapneumovirus, respiratory syncytial virus, and coronaviruses (34, 38). Multiple respiratory viruses can infect the respiratory tract concurrently or sequentially, leading to interactions that may either enhance or suppress the infection and replication dynamics of other viruses (12, 15, 16, 37, 39, 40).

This study aimed to assess trends in respiratory virus circulation among symptomatic individuals presenting to triage areas at our institution and to identify factors associated with the development of severe acute respiratory infections before and after the COVID-19 pandemic.

## 2 Materials and methods

### 2.1 Ethics statement

The study was evaluated and approved by the Research Committee of the Ministry of Health of Jalisco and registered in the State Research Registry under identifier 73/LESP/JAL/2024. Additionally, it was reviewed and approved by the “Comité de Ética en Investigación en Ciencias de la Salud del Centro Universitario de Tlajomulco, Universidad de Guadalajara” (ethical approval number CUTLAJO/DS/CEICS/017/24). This study, involving human participants, was conducted in accordance with the principles of the Declaration of Helsinki (1964) and its subsequent amendments, as well as applicable national legislation and institutional guidelines. As the research was retrospective and exclusively used de-identified data, the requirement for informed consent was waived.

### 2.2 Setting

This study was conducted at OPD Hospitales Civiles de Guadalajara, a tertiary referral and teaching institution affiliated with the University of Guadalajara, located in Jalisco, Mexico. The institution encompasses two referral hospitals that primarily serve an uninsured population from western Mexico, providing specialized care for both adults and children. As major regional centers, these hospitals receive patients from diverse urban and rural areas. During the COVID-19 pandemic, they played a pivotal role in the region by delivering comprehensive general and critical care services while managing a substantial portion of COVID-19 cases.

### 2.3 Population and eligibility criteria

The study population comprised symptomatic patients presenting with influenza-like illness (ILI) to the triage areas of our institution, who were tested for respiratory viruses between January 2018 and March 2024. A suspected case of ILI was defined according to local guidelines as the sudden onset of symptoms accompanied by at least one systemic symptom—fever, feverishness, cough, or headache—and at least one localized symptom, such as dyspnea, myalgias, arthralgias, odynophagia, chills, chest pain, rhinorrhea, tachypnea, anosmia, dysgeusia, or conjunctivitis (41).

Medical and state laboratory records were cross-referenced with the epidemiological surveillance platform to ensure data consistency. Demographic information, comorbidities, and severe acute respiratory infection (SARI) status—defined per local guidelines as a respiratory tract infection accompanied by severe symptoms such as dyspnea, chest pain, acute respiratory distress syndrome, or the need for hospitalization—were systematically recorded (42). Additional variables collected included the number of days from symptom onset to the date of specimen collection for RT-PCR testing, vaccination status, RT-PCR Ct values of detected viruses, and other relevant clinical parameters.

Patients were excluded if their tests did not correspond to symptomatic cases, such as those performed for contact tracing, or if discrepancies were identified between the state surveillance platform and hospital records. Cases requiring subsequent testing to confirm negativity for ending isolation were also excluded. Additionally, records with more than 10% missing sociodemographic or clinical data were omitted.

### 2.4 Viral testing

Nasopharyngeal swabs were collected in viral transport media and transported to the State Public Health Laboratory of Jalisco (SPHLJ) under strict cold chain conditions. Upon arrival, laboratory procedures included viral inactivation, nucleic acid extraction, and viral gene amplification using RT-PCR. The viral genetic material was extracted using automated platforms that employed magnetic bead-based technology to selectively bind viral RNA, followed by sequential washing steps for isolation. Two different extraction kits and systems were utilized for this process: the ExiPrep™ Plus Viral DNA/RNA Kit, designed for 96 reactions and operated on the ExiPrep™ 96 platform (Bioneer^®^, Daejeon, South Korea), and the MagNA Pure 96 Small Volume Kit, used on the MagNA Pure 96 System (Roche^®^, Basel, Switzerland). Viral detection was carried out using Health Mexico-approved single and multiplex RT-qPCR assays. These assays targeted key SARS-CoV-2 genes, including *E, N*, *RdRP*, and *ORF1ab*. Detection platforms and assays employed included the COBAS 6800 System (Roche^®^), the Logix Smart RT–PCR Kit (Co-Diagnostics^®^), the Flu-COVID Vitro Kit (Master Diagnóstica^®^), and the BioFire FilmArray Respiratory Panel (BioFire Diagnostics^®^). The respiratory viruses of interest included SARS-CoV-2, Influenza A virus, Influenza B virus, Respiratory syncytial virus (RSV), Human parainfluenza viruses 1–4 (HPIV1, HPIV2, HPIV3, HPIV4), Human metapneumovirus (hMPV), seasonal Human coronaviruses (HCoV-229E, HCoV-OC43, HCoV-NL63, and HCoV-HKU1), Human adenovirus (HAdV), Human enterovirus/rhinovirus (HEV/HRV), and Human Bocavirus (HBoV). A case was defined as laboratory-confirmed for each symptomatic case with a respiratory virus RT-PCR positive test.

### 2.5 Testing restrictions

In Mexico, prior to the COVID-19 pandemic, respiratory virus surveillance primarily focused on influenza, but it was significantly enhanced with the onset of COVID-19. According to pre-pandemic local guidelines, only 10% of suspected influenza cases among outpatients and 100% of hospitalized cases were tested for Influenza A and Influenza B viruses. Furthermore, 10% of severe cases that tested negative for influenza were evaluated for other respiratory viruses (43). The COVID-19 health emergency in 2020 prompted expanded monitoring and the implementation of new testing strategies. Comprehensive sampling and testing for SARS-CoV-2, Influenza A, and Influenza B were conducted on all suspected ILI and SARI cases using multiplex RT-PCR. Other respiratory viruses were tested in 10% of severe cases (44).

### 2.6 Definition of time periods

Based on the observed pandemic dynamics in western Mexico, we defined three distinct periods to analyze the frequency and positivity rates of respiratory viruses other than SARS-CoV-2: the pre-pandemic period (January 2018 to February 2020), the pandemic period (March 2020 to December 2021), and the post-pandemic period (January 2022 onward). These periods were designed to capture contrasting trends influenced by the implementation and subsequent relaxation of non-pharmaceutical interventions (NPIs). The pre-pandemic period served as a baseline to evaluate respiratory virus circulation under typical conditions. The pandemic period captured the significant decline in positivity rates driven by the emergence of SARS-CoV-2 and widespread NPIs, while the post-pandemic period documented recovery trends as SARS-CoV-2 cases declined and NPIs were progressively lifted. This framework allowed for a detailed analysis of the disruptions caused by the pandemic and the subsequent recovery in respiratory virus activity.

### 2.7 Statistical analysis

Demographic data were reported as simple relative frequencies. The percentage of positive values was calculated as the number of tests that yielded positive results divided by the total number of tests performed for a given period of time expressed as a percentage for each virus. The normality of the data distribution was assessed using the Shapiro–Wilk test. Pearson’s chi-square test and Fisher’s exact test were used to compare proportions, as appropriate. For comparisons of quantitative variables, Student’s t test and the Wilcoxon–Mann–Whitney test were used for normally and nonnormally distributed data, respectively.

To analyze the temporal trends in viral positivity rates, we applied locally estimated scatterplot smoothing (LOESS) to weekly aggregated data for each respiratory virus. LOESS is a non-parametric regression technique that fits localized polynomial curves to the data, enabling the detection of trends and patterns without assuming a specific functional form. Weekly positivity rates for each virus were calculated as the ratio of positive cases to the total number of tests conducted during each week. To represent the uncertainty in trend estimates, 95% confidence intervals were calculated around the LOESS-adjusted curves. Both LOESS-derived positivity rates and raw, unadjusted positivity rates were subsequently used in interrupted time series (ITS) analyses, providing a robust assessment of the immediate and sustained impacts of the COVID-19 pandemic on viral circulation patterns.

To evaluate the impact of the COVID-19 pandemic on respiratory viruses circulation from 2017 to 2023 we use ITS analysis. Segmented linear regression models were used to assess changes in virus activity, with predictors including time, level changes, and trend changes for each period. The analysis was conducted on two distinct datasets: one containing raw weekly positivity rates and another with weekly LOESS-adjusted positivity rates. Both datasets represented positivity trends for influenza, RSV, HEV/HRV, HPIV, HCoV, HAdV, hMPV, and HBoV, aggregated by epidemiological week.

For both approaches, three temporal periods were defined:

1. Pre-pandemic period: Weeks before March 2020.
2. Pandemic period: Weeks between March 2020 and December 2021.
3. Post-pandemic period: Weeks from January 2022 onwards.

The ITS models included the following parameters:

- Constant (*β*₀): Baseline positivity rate at the start of the pre-pandemic period.
- Time (β₁): Trend in positivity rates during the pre-pandemic period.
- Level (*β*₂):
  - Immediate change in positivity rates during the pandemic (2020–2021).
  - Immediate change in positivity rates during the post-pandemic period (2022).
- Trend (β₃):
  - Change in trend during the pandemic (2020–2021).
  - Change in trend during the post-pandemic period (2022).

Separate linear regression models were fitted for each virus using the formula:


$$Y_{t}=\beta_{0}+\beta_{1}{\mathrm{Time}}_{t}+\beta_{2}{\mathrm{Level}}_{t}+\beta_{3}{\mathrm{Trend}}_{t}+\varepsilon_{t}$$

where ***Y****_t_* represents the positivity rate (raw or LOESS-adjusted) for a given virus at time *t*.

The LOESS-adjusted dataset was used to reduce week-to-week variability and better capture long-term trends, while the raw dataset allowed for the analysis of unprocessed data to ensure robustness of findings. Statistical significance for all model parameters (*β* coefficients) was assessed, and results were presented with corresponding 95% confidence intervals, standard errors, and *p*-values.

Given the predominant impact of certain viruses on pediatric populations, the ITS analysis was also stratified by age into two primary groups: <18 years and < 5 years. These cutoffs were chosen to ensure an adequate number of observations in each subgroup, allowing for a reliable model fit. Additionally, ITS analyses of raw SARI proportions were conducted for the general population as well as for four specific age-stratified groups: <5 years, 5–18 years, 18–65 years, and > 65 years. This approach enabled the assessment of pre- and post-pandemic changes in SARI proportions and the detection of potential shifts across different age cohorts. The LOESS-adjusted dataset was primarily used to reduce week-to-week variability and better capture long-term trends, whereas the raw dataset was analyzed to preserve unprocessed data, ensuring the robustness of the findings. Statistical significance for all model parameters (𝛽 coefficients) was evaluated, with results presented alongside 95% confidence intervals, standard errors, and *p*-values.

Finally, multivariate logistic regression was performed to determine independent factors associated with SARI. Variables were included in the model if they met a significance threshold of *p* < 0.1 in univariate analysis and were considered biologically plausible. A stepwise selection method was applied to optimize the model. Model fit was assessed using the Hosmer–Lemeshow test, with *p*-values >0.1 indicating an adequate fit. Confidence intervals were set at 95% (CI = 95%). The final models for each analyzed period were selected based on their goodness of fit, as determined by the Hosmer–Lemeshow test, and explanatory power, as indicated by McFadden’s R^2^ values. The results reflect the best-fitting models for each period and population group studied.

Statistical analyses were conducted using R version 4.3.1 and Python version 3.10. In R, *lm* and *segmented* packages were used for ITS analyses, while LOESS adjusting was applied with *ggplot2* and *stats*. Logistic regression models were implemented using *glm*, supported by *broom* and *ResourceSelection* for model evaluation. In Python, ITS analyses utilized the *statsmodels* library (version 0.14.0) for time-series modeling, with *scipy* (version 1.10.1) aiding hypothesis testing and diagnostics. These tools enabled comprehensive evaluation of virus circulation, positivity trends, and associations with SARI across time periods.

## 3 Results

**Figure 1:**
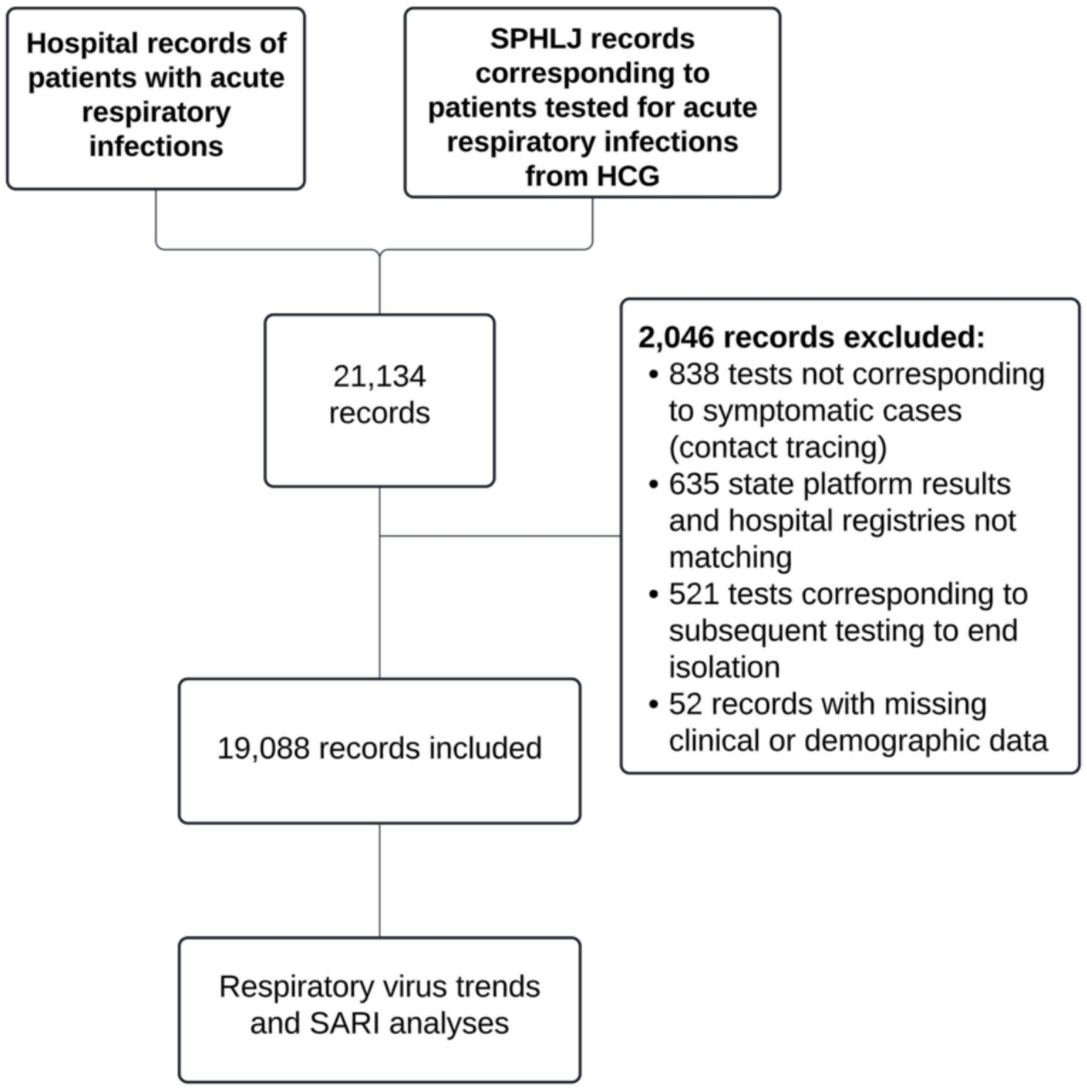
Flowchart of case selection.

From the records reviewed, a total of 21,134 patients were identified, of whom 19,088 met the selection criteria and were included in the analysis, as depicted in Figure 1.

**Figure 2:**
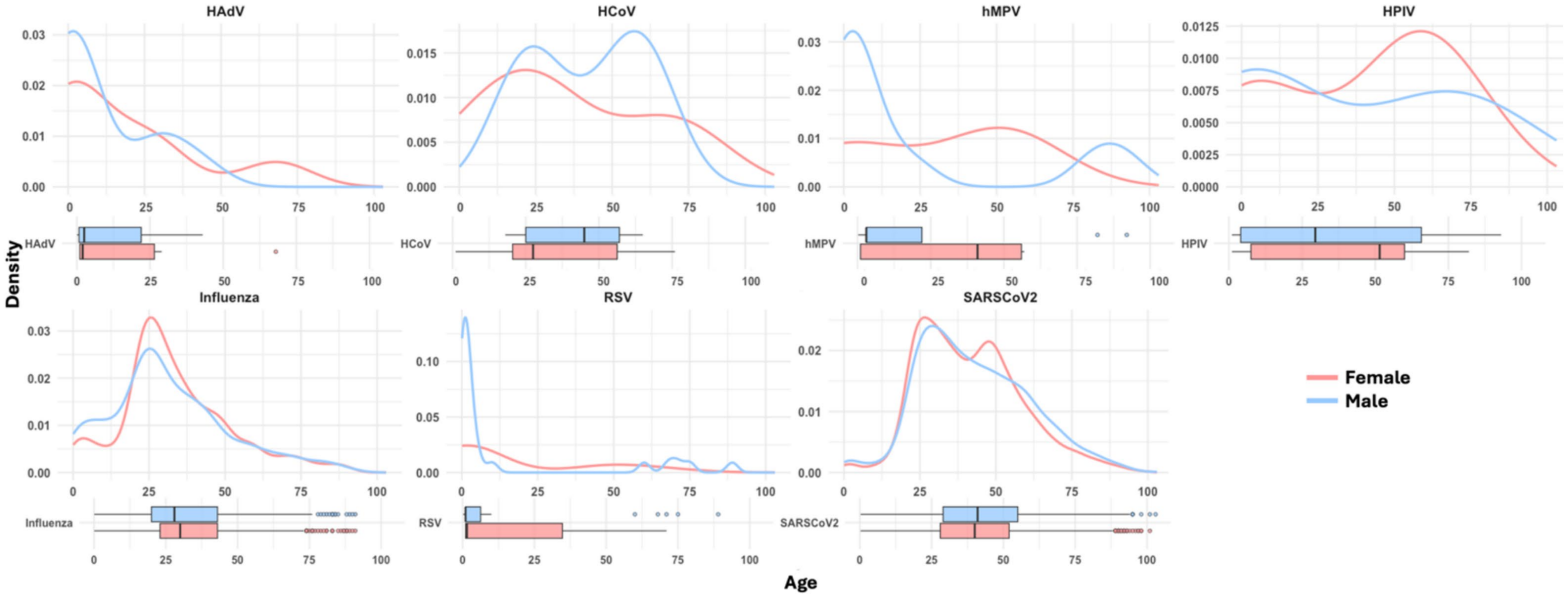
Age and sex distributions of patients by detected respiratory virus.

The median age of the included patients was 36 years (IQR 26–51), with 56.74% (*n* = 10,831) being women. Among patients who tested positive for respiratory viruses, the median age was 38 years (IQR 27–52). The age and sex distributions by detected virus are presented in Figure 2, while additional sociodemographic data and characteristics of the study population are detailed in Table 1.

**Table 1:** Sociodemographic and clinical characteristics of patients tested for respiratory viruses during the study period.

Variable	Total (*n* = 19,088)	Women (*n* = 10,831)	Men (*n* = 8,256)	*p* value
Age—median, (IQR)	36.0 (26.0–51.0)	36.0 (26.0–50.0)	36.0 (25.0–52.0)	0.210
Days of symptoms to test -median, (IQR)	2.0 (1.0–3.0)	2.0 (1.0–3.0)	2.0 (1.0–3.0)	<0.001
Severe acute respiratory infection (SARI)—n, (%)	3,750 (19.65)	1721 (45.89)	2029 (54.11)	<0.001
Comorbidities—n, (%)	6,713 (35.17)	3,604 (33.27)	3,109 (37.66)	<0.001
Diabetes	1859 (9.74)	1,021 (9.43)	838 (10.15)	0.027
Cardiovascular disease	411 (2.15)	219 (2.02)	192 (2.33)	0.092
Obesity	1,377 (7.21)	768 (7.09)	609 (7.38)	0.218
Asthma	716 (3.75)	483 (4.46)	233 (2.82)	<0.001
COPD	368 (1.93)	190 (1.75)	178 (2.16)	0.025
Smoking	1,067 (5.59)	378 (3.49)	689 (8.35)	<0.001
Hypertension	2,316 (12.13)	1,259 (11.62)	1,057 (12.80)	0.002
Immunosuppression status	821 (4.30)	345 (3.19)	475 (5.75)	<0.001
Chronic kidney disease	447 (2.34)	187 (1.73)	260 (3.15)	<0.001
Pregnancy	66 (0.35)	66 (0.61)	0 (0.00)	-

Among the positive tests for respiratory viruses, 36.44% corresponded to SARS-CoV-2 (*n* = 6,783), 12.20% corresponded to influenza virus (*n* = 1,187), and 11.33% to other respiratory viruses distinct from SARS-CoV-2 and influenza (*n* = 175). Regarding influenza subtypes, Influenza A H3 was the most prevalent (*n* = 737), followed by the Influenza B Victoria lineage (*n* = 285) and Influenza A H1N1 (*n* = 111). Among other respiratory viruses (OVRs), Human enterovirus/rhinovirus was the most frequently isolated (*n* = 73), followed by respiratory syncytial virus (*n* = 46). The remaining viral isolates are summarized in Table 2.

**Table 2:** Proportions of viruses detected among patients tested during the study period.

Virus	Total (*n* = 18,616)	Women (*n* = 10,601)	Men (*n* = 8,015)	*p* value
SARSCoV2 infection—n, (%)	6,783 (36.44)	3,915 (36.93)	2,868 (35.78)	0.111
	Total (*n* = 9,726)	Women (*n* = 5,546)	Men (*n* = 4,180)	*p* value
Influenza viruses—n, (%)	1,187 (12.20)	707 (12.75)	480 (11.48)	0.059
Influenza A	879 (9.04)	535 (9.65)	344 (8.23)	0.016
Influenza A H3	737 (7.58)	453 (8.17)	284 (6.79)	0.011
Influenza A H1N1	111 (1.14)	61 (1.10)	50 (1.20)	0.658
Influenza A nonsubtyped	31 (0.32)	21 (0.38)	10 (0.24)	0.227
Influenza B	308 (3.17)	172 (3.10)	136 (3.25)	0671
Influenza B Victoria lineage	285 (2.93)	156 (2.81)	129 (3.09)	0.429
Influenza B Yamagata lineage	15 (0.15)	13 (0.23)	2 (0.05)	0.020
Influenza B nonsubtyped	8 (0.08)	3 (0.05)	5 (0.12)	0.302
	Total (*n* = 1,545)	Women (*n* = 735)	Men (*n* = 810)	*p* value
Other respiratory viruses (ORVs)—n, (%)	175 (11.33)	78 (0.72)	97 (11.98)	0.399
Human adenovirus	17 (1.10)	7 (0.95)	10 (1.23)	0.595
Human metapneumovirus	14 (0.91)	5 (0.68)	9 (1.11)	0.372
Human enterovirus/rhinovirus	73 (4.72)	32 (4.35)	41 (5.06)	0.512
Human parainfluenza virus 1	3 (0.19)	2 (0.27)	1 (0.12)	0.607
Human parainfluenza virus 2	1 (0.06)	1 (0.14)	0 (0.00)	0.961
Human parainfluenza virus 3	11 (0.71)	5 (0.68)	6 (0.74)	0.888
Human parainfluenza virus 4	3 (0.19)	2 (0.27)	1 (0.12)	0.607
Respiratory syncytial virus	46 (2.98)	22 (2.99)	24 (2.96)	0.972
Human bocavirus	2 (0.13)	1 (0.14)	1 (0.12)	1
Human coronavirus 229E	2 (0.13)	1 (0.14)	1 (0.12)	1
Human coronavirus HKU1	3 (0.19)	1 (0.14)	2 (0.25)	1
Human coronavirus NL63	5 (0.32)	1 (0.14)	4 (0.49)	0.377
Human coronavirus OC43	5 (0.32)	3 (0.41)	2 (0.25)	0.673
Viral Coinfections—n, (%)	116 (7.51)	75 (10.20)	41 (5.06)	0.011
Coinfections between SARS-CoV-2 and Influenza	94 (6.08)	63 (8.57)	31 (3.83)	0.059
Coinfections between ORVs (excluding SARS-CoV-2 and Influenza)	14 (0.91)	7 (0.95)	7 (0.86)	0.855
Coinfections between SARS-CoV-2 and ORVs	5 (0.32)	3 (0.41)	2 (0.25)	0.679
Coinfections between Influenza and ORVs	3 (0.19)	2 (0.27)	1 (0.12)	0.607

**Figure 3:**
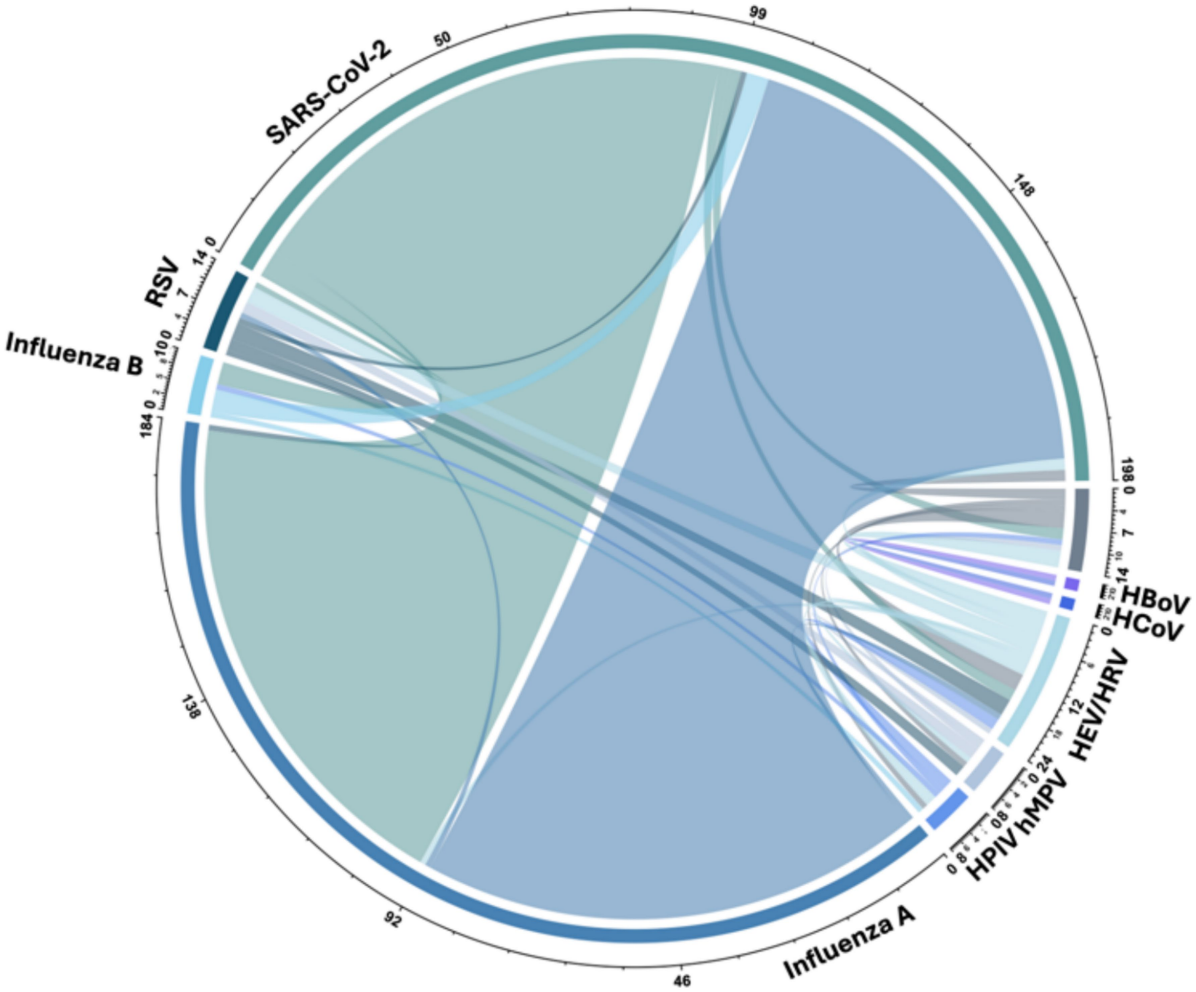
Coinfections among detected respiratory viruses.

**Figure 4:**
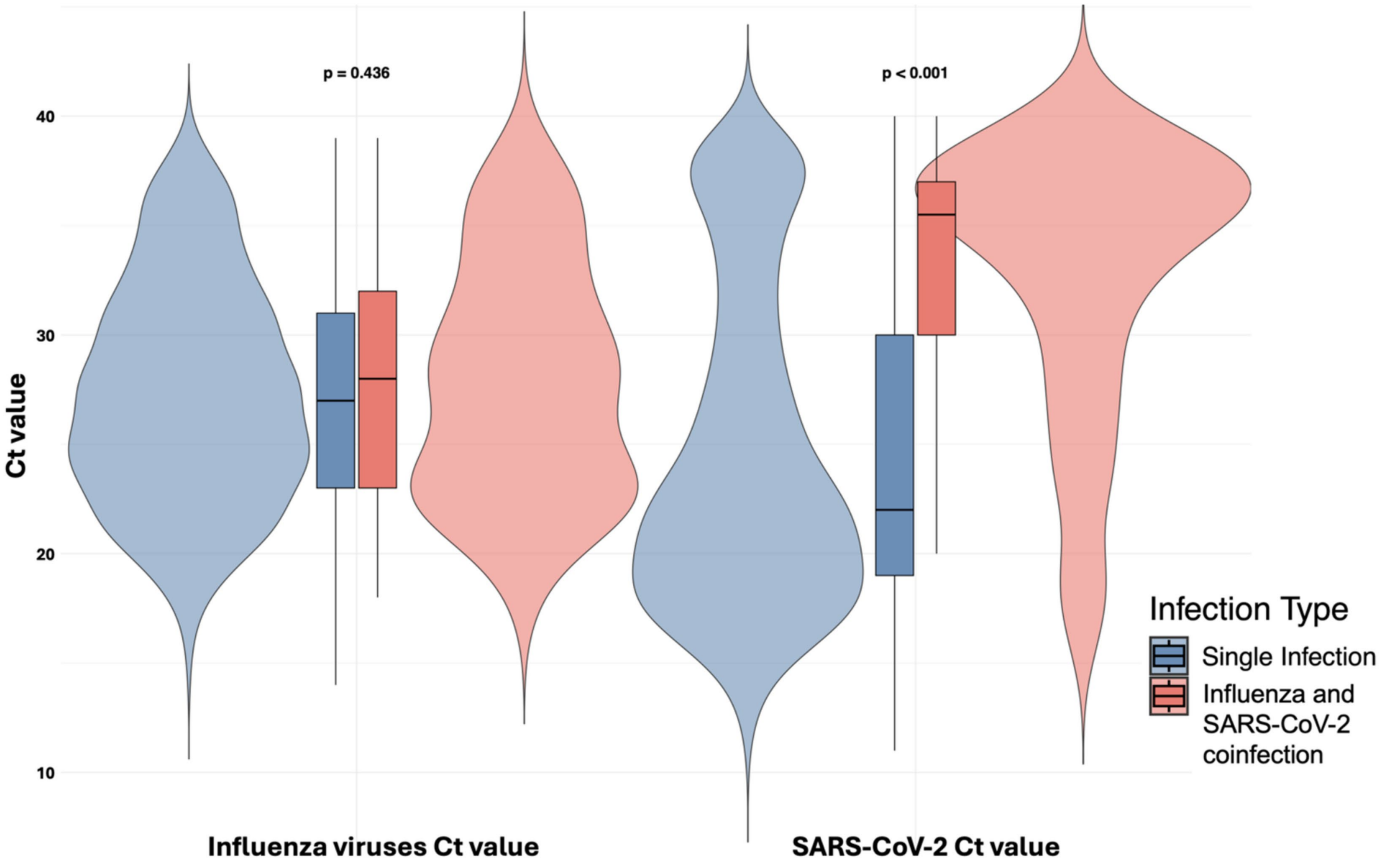
Comparison of Ct values in single infections versus influenza and SARS-CoV-2 coinfections.

A total of 116 respiratory virus coinfections were identified. Among these, 94 cases involved coinfections between SARS-CoV-2 and influenza, 14 cases were coinfections among respiratory viruses other than SARS-CoV-2 and influenza, 5 cases were coinfections between SARS-CoV-2 and other respiratory viruses distinct from influenza, and 3 cases involved coinfections between influenza and other respiratory viruses distinct from SARS-CoV-2. Notably, SARS-CoV-2 Ct values were higher (indicating lower viral loads) in patients with influenza coinfection (SARS-CoV-2 Ct median: 35.5, IQR 30–37) compared to those without influenza coinfection (SARS-CoV-2 Ct median: 22, IQR 19–30), *p* < 0.001. No significant differences were observed in influenza Ct values between patients with and without SARS-CoV-2 coinfection, with median values of 27 (IQR 23–31) in coinfected patients versus 28 (IQR 23–32) in non-coinfected patients. The detailed distribution of these coinfections is described in Table 2 and illustrated in Figure 3, as well as the distributions of influenza and SARS-CoV-2 Ct values in both coinfected and non-coinfected patients, which are depicted in Figure 4.

**Figure 5:**
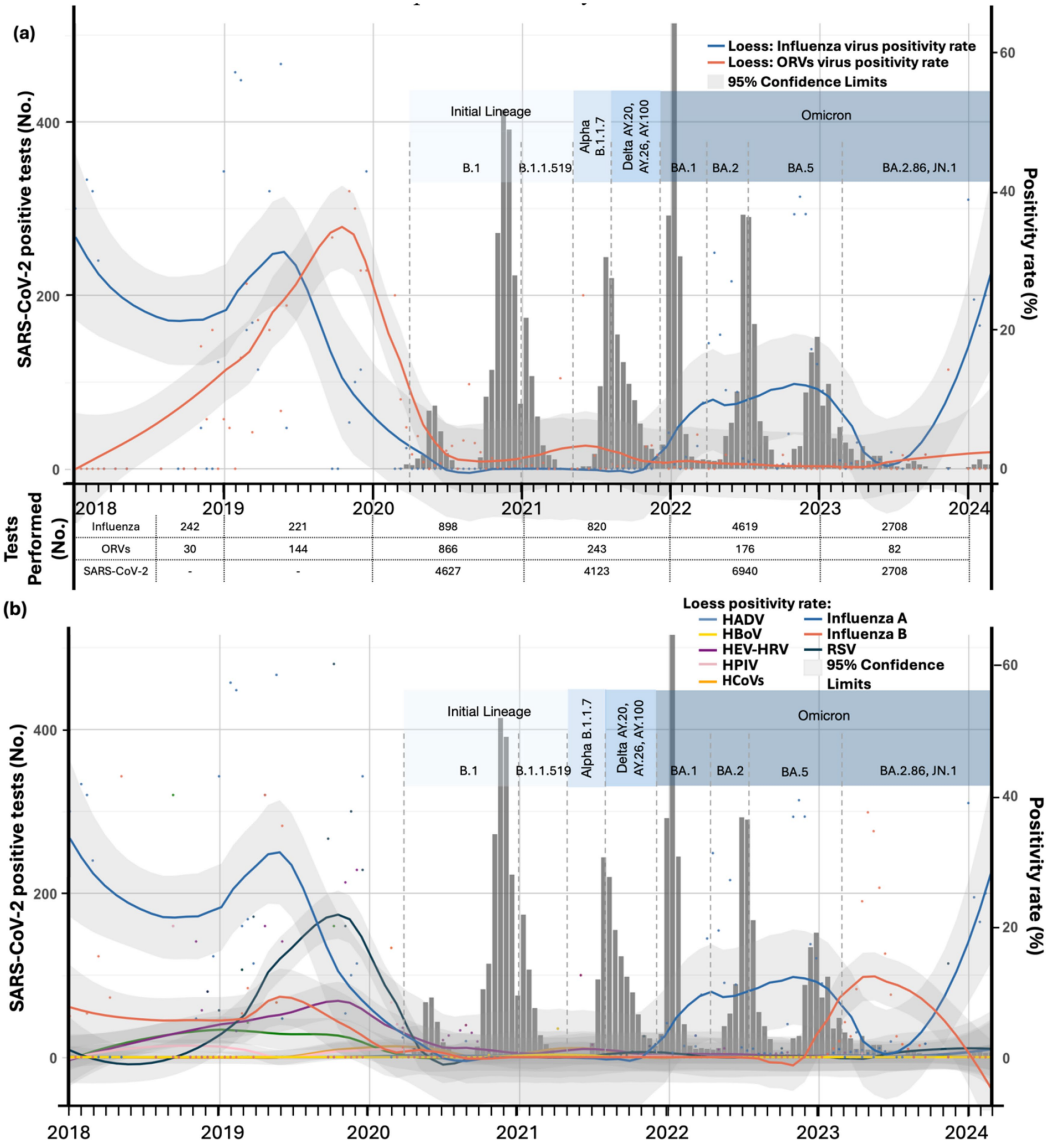
Positivity rates of influenza, other respiratory viruses (ORVs), and SARS-CoV-2 cases across pandemic years. **(A)** Positivity rates of influenza viruses and other respiratory viruses as a group, in relation to SARS-CoV-2 positive cases. **(B)** Positivity rates of influenza virus subtypes and individual respiratory viruses, in relation to SARS-CoV-2 positive cases. Dots represent raw positivity rates, while lines represent LOESS-adjusted positivity rates with 95% confidence intervals. The figure includes key SARS-CoV-2 variants detected during the timeline, contextualized with the number of tests performed each year.

Figure 5 illustrates the positivity rates of influenza, other respiratory viruses, and SARS-CoV-2 cases across pandemic years. Regarding influenza viruses, a significant reduction in positivity rates was observed during the first year of the COVID-19 pandemic. However, following 2020, and particularly after 2021, a marked increase in the positivity rates of influenza viruses and their subtypes was documented (Figures 5A,B). Notably, in 2023, after the rise in positivity rates for influenza A during 2022, an increase in the positivity rate of the influenza B Victoria lineage was observed (Figure 5B). Conversely, the Influenza B Yamagata lineage remained completely undetected during and after the pandemic.

For other respiratory viruses, positivity rates as a group declined to historically low levels during the 3 years following the onset of the pandemic (Figure 5A). Individually, however, certain viruses such as HEV/HRV and HPIV continued to be detected, and a slight increase in RSV positivity was noted in the last quarter of 2021 (Figure 5B). Despite this, all these viruses remained at historically low levels and had not returned to pre-pandemic rates as of the time of this report.

Utilizing LOESS-adjusted positivity rates, the ITS analysis revealed significant disruptions in the positivity rates of respiratory viruses during the COVID-19 pandemic (2020–2021), with varied recovery patterns post-2022. Influenza experienced a sharp and significant immediate decline in positivity during 2020–2021 (Level β2 2020–2021: −44.166, *p* < 0.001), followed by a further reduction in 2022 (Level β2 2022: −51.761, *p* < 0.001), despite a slight upward recovery trend post-2022 (Trend β3 2022: 0.1061, *p* < 0.001). RSV showed a significant immediate increase during the pandemic (Level β2 2020–2021: 1.198, *p* = 0.020), with a non-significant decrease in 2022 (Level β2 2022: −0.0842, *p* = 0.900) but a significant upward trend post-2022 (Trend β3 2022: 0.0072, *p* = 0.031). HEV/HRV exhibited a substantial increase during the pandemic (Level β2 2020–2021: 5.5102, *p* < 0.001), followed by a significant downward trend (Trend β3 2020–2021: −0.0455, *p* < 0.001). HCoV and HAdV also showed significant increases during the pandemic (Level β2 2020–2021: 1.4338 and 0.4478, respectively, both *p* < 0.001), with HCoV demonstrating a declining trend post-pandemic (Trend β3 2020–2021: −0.0108, *p* < 0.001) and HAdV exhibiting a reduction post-2022. hMPV had significant increases during 2020–2021 (Level β2 2020–2021: 1.1036, *p* < 0.001) but showed limited recovery post-pandemic. Conversely, HPIV and HBoV exhibited minimal changes during the pandemic and subsequent periods, with trends remaining stable. A summary of the ITS results for both LOESS-adjusted positivity rates and raw positivity rates is provided in Table 3, while an extended version can be found in Supplementary Table S1.

**Table 3:** Interrupted time series analysis using LOESS positivity rates for influenza, respiratory syncytial virus, human enterovirus/rhinovirus, and human adenovirus during the pre-pandemic, pandemic (2020–2021), and post-pandemic periods (2022–2023).

	Influenza			Respiratory syncytial virus			Human enterovirus/rhinovirus			Human adenovirus		
Variable	*β*	SE	*p* value	*β*	SE	*p* value	*β*	SE	*p* value	*β*	SE	*p* value
Constant (β0)	45.32	1.04	<0.001	−0.96	0.29	<0.001	−0.28	0.17	0.098	−0.05	0.03	0.0840
Time (β1)	−0.60	0.04	<0.001	0.20	0.01	<0.001	0.16	0.01	<0.001	0.01	0.01	0.0030
Level												
2020–2021 (β2)	−44.16	1.860	<0.001	1.20	0.51	0.020	5.51	0.31	<0.001	0.45	0.05	<0.001
2022 (β2)	−51.76	2.44	<0.001	−0.08	0.67	0.900	0.87	0.40	0.030	−0.25	0.07	<0.001
Trend												
2020–2021 (β3)	−0.01	0.02	0.566	0.01	0.01	0.840	−0.05	0.01	<0.001	−0.01	0.01	<0.001
2022 (β3)	0.11	0.01	<0.001	0.01	0.01	0.031	−0.01	0.01	0.448	0.01	0.01	<0.001

The ITS analysis utilizing the raw positivity rates revealed noticeable differences in the significance and magnitude of the trends and levels observed during the pandemic (2020–2021) and post-pandemic periods (2022 onwards). For influenza, raw positivity rates only indicated a smaller and non-significant decline during 2020–2021 (β2: −3.63, *p* = 0.513) and a significant increase in 2022 (β2: 14.81, *p* = 0.033). For RSV, raw positivity rates highlighted a sharper initial decline during 2020–2021 (β2: −0.85, *p* = 0.004) and no significant trend post-2022 (β3: 0.00, *p* = 0.771). HEV/HRV displayed a substantial increase during 2020–2021 in both analyses. Other viruses, such as HCoV and HAdV, also showed marked differences. For HBoV, the ITS analysis could not be performed using raw positivity rates due to insufficient data points. LOESS-adjusted rates captured more pronounced shifts, particularly in post-pandemic recovery trends, which were less evident or not significant in the raw data. The complete ITS analyses utilizing LOESS-adjusted and raw positivity rates can be found in Supplementary Table S1.

In children under 5 years of age, influenza initially presented at a high baseline (~28.8%, *p* = 0.003) and exhibited two significant declines: one at the onset of the pandemic (Level *β*₂ = −23.23, *p* = 0.045) and another in early 2022 (−24.18, *p* = 0.020). In contrast, HEV/HRV demonstrated a positive pre-pandemic trend (+0.19 per week, *p* = 0.041) but remained largely unaffected during the pandemic and in 2022. RSV, however, showed a significant increase in slope following the pandemic’s onset (+0.26 per week, *p* < 0.001) without subsequent shifts. Other respiratory viruses (ORVs), including HPIV and HCoV, displayed minimal or no significant changes in children under five, aside from a borderline increase in the pandemic slope for ORVs (*p* = 0.057). Among older children and adolescents (<18 years), influenza—despite its notable decline in younger children—did not exhibit clear changes, while RSV accelerated an already increasing trend (*p* = 0.001). HEV/HRV, ORVs as a group, and HPIV demonstrated limited or no immediate pandemic-related alterations, although some displayed positive slopes before and/or during the pandemic. Overall, RSV experienced the most pronounced increase in both pediatric subgroups, whereas influenza reductions were primarily observed in children under five. The complete ITS analysis with LOESS-adjusted trends stratified by age in non-adult populations is available in Supplementary Table S2.

In the overall population, our interrupted time series (ITS) analysis of SARI proportions demonstrated a high baseline at the study’s outset, coupled with a progressive pre-pandemic increase. Specifically, during the pre-pandemic phase (before March 2020), the baseline (*β*₀) was approximately 43% (*p* < 0.001) and the weekly slope (Time, β₁) rose by +0.49 points (*p* < 0.001), indicating a steady, moderate growth in SARI prior to the pandemic. Upon entering the pandemic period (March 2020–December 2021), no significant immediate change (Level, β₂) was detected (*p* = 0.789), suggesting that the pandemic did not produce a sharp, abrupt shift in SARI at its onset. Nonetheless, there was a statistically significant reduction in the slope (Trend, β₃ ≈ −0.21, *p* = 0.008), implying that, although SARI continued to rise, it did so at a slower rate compared to pre-pandemic levels. In January 2022, marking the post-pandemic phase, SARI exhibited a pronounced drop of approximately 37 percentage points (*p* < 0.001), followed by a renewed weekly uptick of +0.33 points (*p* < 0.001). Thus, after an abrupt decline at the onset of 2022, SARI subsequently rebounded with a positive trend in the post-pandemic period. Overall, these findings suggest that while SARI was already climbing in the months leading up to COVID-19, the pandemic brought about a significant slowing of that trajectory. Despite no immediate “jump” at pandemic onset, a sharp decrease occurred as public health measures and viral circulation patterns evolved at the start of 2022. Notably, this decline was then followed by an upswing, reflecting the dynamic interplay of shifting viral landscapes, evolving interventions, and possible changes in healthcare-seeking or diagnostic practices post-pandemic. The detailed ITS analysis of SARI proportions, including trend estimates and statistical findings, is presented in Table 4.

**Table 4:** Interrupted time series analysis of SARI proportions, during the pre-pandemic, pandemic (2020–2021), and post-pandemic periods (2022–2023).

Variable	General population			Age group < 5 years			Age group 5–18 years			Age group 18–65 years			Age group > 65 years		
	*β*	SE	*p* value	*β*	SE	*p* value	*β*	SE	*p* value	*β*	SE	*p* value	*β*	SE	*p* value
Constant (β0)	43.26	5.83	<0.01	77.8	18.65	<0.01	52.03	11.36	<0.01	33.96	8.00	<0.01	29.85	5.48	<0.01
Time (β1)	0.49	0.12	<0.01	−0.12	0.80	0.88	0.21	0.28	0.45	1.28	0.32	<0.01	–	–	–
Level															
2020–2021 (β2)	1.93	7.19	0.79	−31.17	22.60	0.17	−15.98	14.04	0.26	5.29	9.04	0.56	45.30	7.72	<0.01
2022 (β2)	−37.45	7.09	<0.01	−27.35	20.26	0.18	−14.40	12.96	0.27	−34.73	8.90	<0.01	–	–	–
Trend															
2020–2021 (β3)	−0.21	0.08	0.01	0.00	0.34	0.99	0.08	0.17	0.65	−0.21	0.08	0.01	−0.26	0.10	<0.01
2022 (β3)	0.33	0.07	<0.01	−0.09	0.19	0.64	0.04	0.15	0.80	0.31	0.07	<0.01	0.43	0.10	<0.01

– Parameters were not estimable due to insufficient data points or collinearity during those time segments. For older adults (>65 years), the number of weekly observations in the pre-pandemic and post-2022 periods was limited, thus preventing stable estimation of the slope (Time) and level shift (Level) parameters in those intervals.

Across the four age-stratified groups, SARI proportions displayed distinct patterns. In children under 5 years (<5), the baseline was notably high at nearly 78% (*p* < 0.001), yet neither the onset of the pandemic nor the post-pandemic period revealed significant immediate or trend-related changes. By contrast, in older children (5–18 years), the estimated intercept was about 52% (*p* < 0.001) but likewise showed no meaningful shifts during or after the pandemic; any modest tendencies did not reach statistical significance. Among adults aged 18–65 years, a baseline of approximately 34% (*p* < 0.001) was accompanied by a strong pre-pandemic slope (+1.28/week, *p* < 0.001), followed by a slight (and non-significant) level change during the pandemic but a pronounced drop (−35 points, *p* < 0.001) and subsequent rebound (+0.31/week, *p* < 0.001) at the start of 2022. Lastly, in older adults (>65 years), the model indicated a lower initial estimate (~30%, *p* < 0.001) but with a marked immediate jump of +45 points when the pandemic began (*p* < 0.001), coupled with a mild negative slope thereafter (−0.26/week, *p* = 0.012); however, the post-2022 level could not be estimated due to insufficient data, even though the trend in that phase became significantly positive (+0.43/week, *p* < 0.001). These age-specific findings suggest that while younger individuals had either persistently high SARI or inconclusive changes, middle-aged adults experienced a sharp 2022 downturn and rebound, and older adults saw the largest pandemic-era surge but a partial tapering over time. The detailed ITS analysis of SARI proportions, including trend estimates and statistical findings across age stratified groups, is also presented in Table 4.

The univariate analysis of factors associated with SARI revealed significant associations in both the pre-pandemic and post-pandemic periods. Predictors such as age, sex, and the presence of comorbidities consistently demonstrated strong associations with SARI across various subgroups. Among these, comorbidities exhibited a particularly robust relationship, with higher proportions of SARI cases observed in patients with pre-existing conditions during both periods. Notably, in the post-pandemic period, the influence of comorbidities was further accentuated, with a markedly higher proportion of SARI cases attributable to these factors. Comprehensive results from the univariate analyses, including stratifications by virus groups for both periods and by age for the post-pandemic period, are presented in Supplementary Table S3.

**Figure 6:**
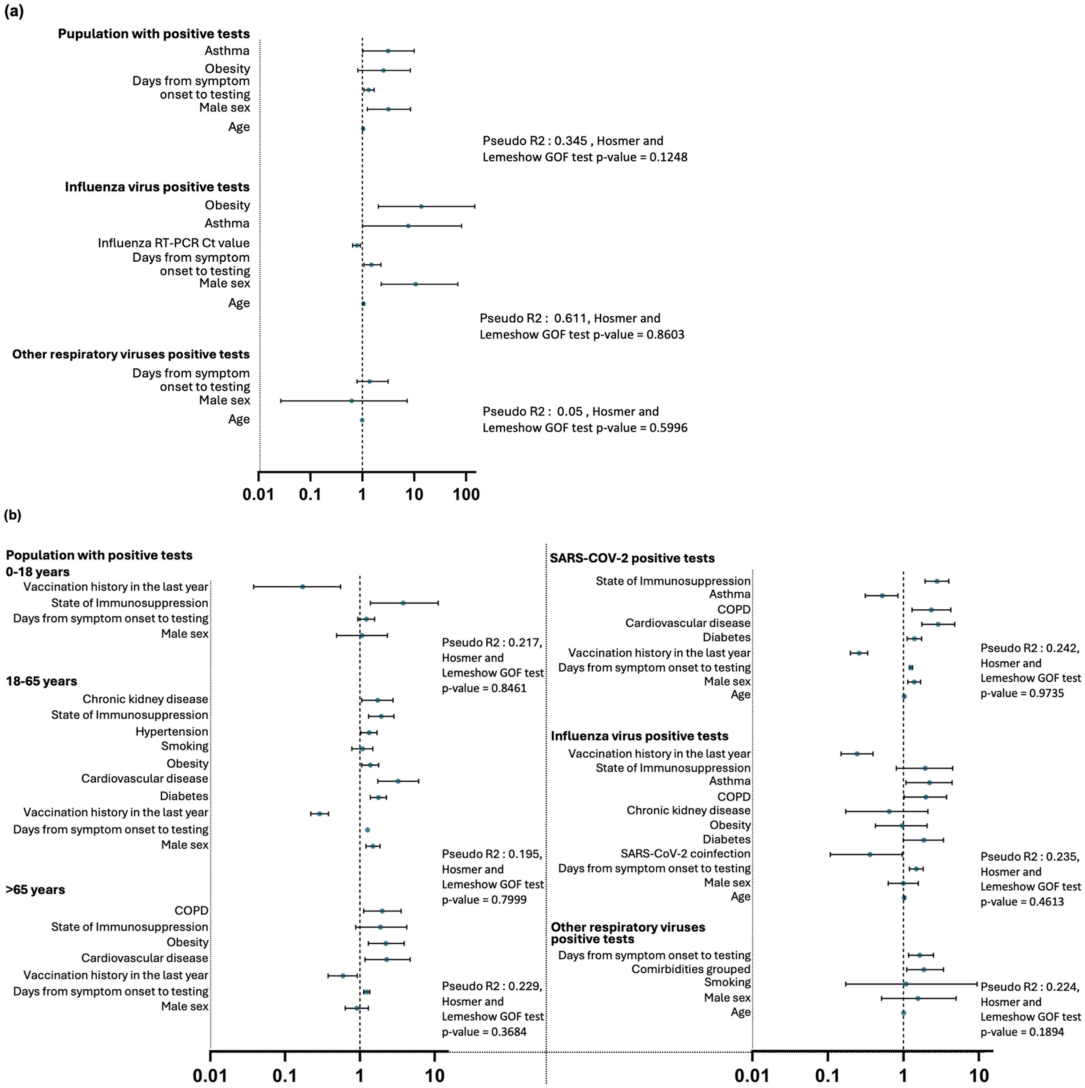
Logistic regression analyses for factors associated with SARI by time periods and virus types. **(a)** Pre-pandemic period analyses. **(b)** Post-pandemic period analyses. *GOF, Goodness-of-Fit.

Figure 6A presents the adjusted odds ratios (ORs) for factors associated with SARI from the logistic regression analyses conducted during the pre-pandemic period. The analysis identified distinct risk patterns among patients with positive test results for any respiratory virus. Significant associations with SARI were observed for age, male sex, and days from symptom onset to testing (*p* < 0.05), with age (OR = 1.04, CI: 1.02–1.06) and male sex (OR = 3.18, CI: 1.25–8.51) emerging as notable risk factors. In the influenza-specific model, Ct values from influenza RT-PCR tests demonstrated a protective effect (OR = 0.786, CI: 0.649–0.916), while obesity was strongly associated with an increased SARI risk (OR = 13.7, CI: 2.03–147.0). Conversely, the analysis of other respiratory viruses (OVRs) revealed no significant associations, with limited case numbers contributing to a low model fit (McFadden’s R^2^ = 0.05), highlighting challenges in assessing ORV-related outcomes. The complete data from the logistic regression models of the pre-pandemic period are detailed in Supplementary Table S4.

Figure 6B presents the adjusted odds ratios (ORs) from the logistic regression analyses of factors associated with SARI during the post-pandemic period. Risk factors for SARI varied significantly across age groups among patients with positive test results for any virus. Among individuals aged 0–18 years, immunosuppression emerged as a significant risk factor (OR = 3.8, CI: 1.38–11.2, *p* = 0.011), while vaccination demonstrated a robust protective effect (OR = 0.172, CI: 0.038–0.554, *p* = 0.008). In adults aged 18–65 years, key risk factors included cardiovascular disease (OR = 3.26, CI: 1.74–6.11, *p* < 0.001), diabetes (OR = 1.77, CI: 1.38–2.27, *p* < 0.001), and obesity (OR = 1.38, CI: 1.06–1.78, *p* = 0.015), while vaccination continued to demonstrate a protective association (OR = 0.291, CI: 0.221–0.381, *p* < 0.001). For individuals aged >65 years, cardiovascular disease (OR = 2.29, CI: 1.17–4.73, *p* = 0.019), obesity (OR = 2.23, CI: 1.30–3.93, *p* = 0.004), and chronic obstructive pulmonary disease (COPD; OR = 1.99, CI: 1.13–3.57, *p* = 0.019) emerged as significant risk factors, while vaccination showed a protective effect (OR = 0.594, CI: 0.377–0.927, *p* = 0.023).

The post-pandemic analysis of specific viral infections revealed further associations with SARI. For SARS-CoV-2, age (OR = 1.03, CI: 1.02–1.03, *p* < 0.001), days from symptom onset to testing (OR = 1.26, CI: 1.20–1.32, *p* < 0.001), cardiovascular disease (OR = 2.88, CI: 1.75–4.79, *p* < 0.001), and immunosuppression (OR = 2.79, CI: 1.93–4.00, *p* < 0.001) were significant risk factors, while vaccination provided robust protection (OR = 0.259, CI: 0.199–0.337, *p* < 0.001). For post-pandemic influenza, days from symptom onset to testing (OR = 1.48, CI: 1.20–1.83, *p* < 0.001) and asthma (OR = 2.22, CI: 1.08–4.42, *p* = 0.026) were associated with SARI, with vaccination again showing a protective effect (OR = 0.244, CI: 0.150–0.397, *p* < 0.001). For OVRs, days from symptom onset to testing (OR = 1.65, CI: 1.17–2.50, *p* = 0.009) and the presence of comorbidities as a group (OR = 1.86, CI: 1.11–3.41, *p* = 0.027) were significant risk factors for SARI. The complete data from the logistic regression models of the post-pandemic period are detailed in Supplementary Table S5.

## 4 Discussion

In our analysis, we observed significant decreases in the circulation of respiratory viruses other than SARS-CoV-2 during the pandemic period, which were tested in our institution, followed by a marked increase in influenza circulation. Additionally, we identified that the main variables associated with an increased risk of developing SARI were sociodemographic factors and comorbidities. This study enhances the limited body of knowledge on respiratory virus trends across pre-pandemic and post-pandemic periods in reference healthcare institutions, while also examining factors associated with SARI development in patients seeking care in a middle-income Latin American country.

Currently, some studies addressing this issue in reference hospitals in middle-income Latin American countries are available in the literature. A report conducted in Brazil in 2021 (45), documented a very low frequency of influenza A among hospitalized patients with ARDS during the COVID-19 pandemic, likely due to preventive measures and high influenza vaccination coverage. Another study, conducted in a pediatric hospital in Argentina (46), reported a decrease in hospitalization rates due to acute lower respiratory tract infections in children, with no seasonal respiratory virus circulation observed during the first year of the pandemic. A third report, also from Brazil (47), examined seasonal respiratory virus trends in pediatric patients, showing a significant increase in the circulation of non-SARS-CoV-2 viruses following the relaxation of pandemic response measures. Other studies conducted in hospital units in developed countries have documented similar trends in respiratory virus circulation, showing significant decreases in positivity and diversity during the pandemic, followed by a gradual resumption of activity in the post-pandemic period. However, these studies did not perform an interrupted time series analysis on the frequency of SARI before, during, and after the COVID-19 pandemic (45–48).

Based on data obtained from our institution, we observed significant reductions in respiratory virus circulation during the pandemic period, with influenza viruses and their subtypes being particularly affected. This was followed by a notable resurgence beginning in 2022. Similarly, other respiratory viruses, distinct from influenza and SARS-CoV-2, experienced marked declines during the pandemic, primarily driven by reductions in respiratory syncytial virus (RSV) and human rhinovirus (HRV) circulation. After 2 years of historically low influenza positivity rates, a significant rebound in influenza viruses and their subtypes was documented. Likewise, other respiratory viruses showed an increase as a group after the first year of the pandemic (Figure 5A), predominantly driven by increases in rhinovirus and respiratory syncytial virus (Figure 5B). Notably, an alternating non-seasonal pattern in influenza virus subtypes emerged from 2022 onward, characterized by fluctuations between Influenza A H3 and Influenza B Victoria lineage.

These findings in our institution also align with population-based studies conducted in other regions of Latin America. For instance, countries like Chile reported historically low influenza activity during the pandemic, characterized by atypical timing and altered duration of influenza seasons (49). For instance, Chile’s influenza season in 2022 began earlier than usual, predominantly with influenza A (H3N2) virus, reflecting a disruption in typical seasonal patterns (49). This disruption was also observed in other Southern Hemisphere countries, where influenza activity decreased significantly in 2020 compared to previous years (50, 51). In Europe, similar trends were noted, with a resurgence of respiratory viruses like influenza and RSV following the relaxation of NPIs (52). In Catalonia, Spain, for example, a resurgence of non-SARS-CoV-2 respiratory viruses was observed during the de-escalation of COVID-19 measures, indicating a shift in the epidemiological patterns of these viruses (52). Asia also experienced changes in respiratory virus activity, with reductions in influenza and RSV infections noted during the pandemic (53). A global analysis of respiratory viral circulation indicated that the pandemic altered the typical patterns of these viruses, leading to earlier peaks and extended durations in some regions (53).

Although post-pandemic increases were observed for both influenza and, to a lesser extent, other respiratory viruses distinct from influenza and SARS-CoV-2, positivity rates recorded at our center have not yet returned to their pre-pandemic levels or seasonal patterns at the time of this report. This phenomenon has been previously documented in a study conducted 6 years after the 2009 H1N1 pandemic in China. In that study, by comparing pre-pandemic, pandemic, and post-pandemic periods, the authors reported that the 2009 H1N1 pandemic influenced the age distribution, seasonal patterns, and peak timing of other respiratory viruses, likely due to immune interference and changes in health-seeking behavior (9).

These findings may reflect mainly the impact of public health measures implemented for infection control and changes in human behavior during the pandemic periods (54). The widespread adoption of NPIs, such as mask-wearing, social distancing, hand hygiene, travel restrictions, and school closures, played a significant role in reducing the transmission of respiratory viruses (55). These measures were initially implemented to control the spread of SARS-CoV-2 but also effectively reduced the transmission of other respiratory pathogens, including influenza and RSV (6). However, viral circulation patterns may not have been solely driven by NPIs; changes in human behavior, such as reduced mobility and increased time spent at home, may have also contributed to the decline in respiratory virus transmission (56). For example, in Hong Kong, a significant reduction in close contacts was observed, which correlated with a decrease in the effective reproduction number of influenza (56). Similar behavioral changes were noted in other regions, further supporting the role of reduced interpersonal interactions in limiting virus spread (57). There is evidence suggesting that viral interference, particularly the presence of SARS-CoV-2, may have contributed to the suppression of other respiratory viruses (57). This interference could be mediated by the host’s immune response, such as the induction of interferons, and immune-mediated interference, modulation of viral replication, competition for cellular entry via ACE2 and neuropilin-1, and dysregulation of the inflammatory response, which can either suppress or enhance the replication of co-circulating viruses (21–25, 57).

The resurgence of respiratory viruses following the COVID-19 pandemic can be attributed to several factors, including the relaxation of NPIs, a decline in natural immunity due to reduced viral circulation during strict public health measures, and a resulting increase in the pool of susceptible individuals (58). Changes in human behavior and testing practices, coupled with heightened public awareness and surveillance, may have also contributed to the increased detection of infections after the most intense phases of the pandemic (59). Additionally, the overall reduction in respiratory virus transmission during the pandemic led to decreased genetic diversity in several viruses, potentially altering their evolutionary dynamics and increasing the likelihood of larger outbreaks once restrictions were lifted (60).

Furthermore, beyond the role of NPIs and behavioral changes, the interplay between host immunity, antigenic drift, and evolutionary pressures on viral strains may have contributed to post-pandemic shifts in viral circulation (61). The limited circulation of certain respiratory viruses during the pandemic likely imposed selective pressures that favored the emergence of antigenic variants, potentially influencing their post-pandemic resurgence (62). Similarly, adaptive immune responses generated by prior infections or vaccinations could have shaped susceptibility patterns and viral competition dynamics (63). Vaccination strategies may have also played a role in shaping viral interactions, as cross-protection and trained immunity from influenza and other vaccines could have temporarily influenced susceptibility patterns and viral competition (64).

Interestingly, our analysis suggested that during both the pre-pandemic and post-pandemic periods, most SARI cases were associated with male sex, the presence of various types of comorbidities, older age, and an increase in the number of days from symptom onset to testing, which may reflect delayed diagnosis. Among these, some associations with SARI are worth highlighting, such as the presence of obesity in influenza cases during the pre-pandemic period; the association with male sex in populations testing positive for any virus, including influenza; immunosuppression status in the 0–18-year age group during the post-pandemic period; the higher association of cardiovascular disease in individuals older than 18 years; the protective effect of vaccination across subgroups; and the association between prolonged symptom duration before testing and SARI in various subgroups. These findings are consistent with previous studies that have highlighted risk factors for the development of SARI, including older age, male sex, comorbidities such as hypertension, diabetes, cardiovascular diseases, chronic respiratory diseases, and immunosuppression status (65–71).

Regarding the increase in days from symptom onset to testing and its relationship with SARI, we believe this finding should be interpreted with caution, as it may not indicate a direct causal relationship. Instead, it could reflect logistical or systemic barriers, such as delays in transportation to healthcare facilities, referral processes from other centers, or internal delays in testing and laboratory processing. This association may serve as a proxy for unmeasured factors, such as disparities in healthcare access or the severity of illness contributing to these delays. Socioeconomic disparities have been shown to affect COVID-19 screening and hospitalization rates. For instance, non-Hispanic Black patients and those from low-income neighborhoods were more likely to test positive for SARS-CoV-2 and were more frequently tested in emergency departments, which is associated with higher hospitalization rate (72). Testing accessibility is another critical factor. Disparities in access to testing can lead to underdiagnosis and delayed treatment of respiratory infections, exacerbating their severity. For example, communities with lower socioeconomic status often face barriers to testing, which can result in higher rates of undiagnosed infections and subsequent severe outcomes (73). Healthcare-seeking behavior is also shaped by socioeconomic factors. Individuals from lower socioeconomic backgrounds may delay seeking care due to financial constraints or lack of access to healthcare facilities, leading to more severe disease presentations when they do seek care (74). Further research is needed to better elucidate the potential implications of this association and the underlying mechanisms driving these delays, particularly in the context of healthcare access disparities, testing availability, and systemic barriers that may contribute to more severe disease presentations.

Although not statistically significant according to the multivariate models with the highest level of adjustment possible, co-infection with SARS-CoV-2 exhibited a trend toward a negative association in cases of influenza during the post-pandemic period. This finding contrasts with studies suggesting that co-infection with SARS-CoV-2 and influenza may increase disease severity (75). Conversely, other studies propose that co-infection does not exacerbate severity and may even mitigate it through mechanisms involving viral or immunological interference (11, 20, 76–78). However, it is important to acknowledge the inherent limitations of our study, which relies on data from a single institution rather than systematic, community-level surveillance. The study population comprises patients who sought care, and as such, it does not represent the broader community. The higher Ct values observed in SARS-CoV-2 among patients co-infected with influenza, indicating lower viral loads, could be due to competitive interactions between the two viruses. The presence of influenza virus might limit SARS-CoV-2 replication, either through direct viral interference or by triggering innate immune responses that modulate viral load dynamics (11). Previous studies have suggested that influenza virus infection may induce robust interferon-mediated antiviral responses, which could suppress SARS-CoV-2 replication in co-infected individuals (11). However, the specific mechanisms underlying this interaction remain unclear and warrant further investigation. Additionally, the testing volume from our institution alone is insufficient to draw definitive conclusions about viral interference, and this potential phenomenon was not studied at the individual level, falling beyond the scope of this study. Nevertheless, these findings underscore the need for further research to better understand the potential implications and mechanisms of viral interactions.

Another noteworthy observation was the behavior of asthma as a factor associated with SARI in patients with influenza during the post-pandemic period, which, in contrast, demonstrated a negative association with SARI in cases of confirmed SARS-CoV-2. While chronic pulmonary diseases have traditionally been linked to poor outcomes in patients with viral respiratory infections, the current consensus in the medical literature suggests that asthma does not significantly increase the risk of severe outcomes from COVID-19 for most patients (79, 80). Certain aspects of asthma may influence the course of SARS-CoV-2 infections. For example, research has shown that specific asthma phenotypes, such as allergic asthma, may have a protective effect, with allergic asthmatics being less likely to require hospitalization compared to non-allergic asthmatics (81). Additionally, some studies suggest that certain asthma treatments, such as inhaled corticosteroids, may confer a protective effect against severe COVID-19 outcomes (82). However, a systematic review indicated that adults with severe asthma, particularly those requiring high-dose inhaled corticosteroids or oral corticosteroids, face a higher risk of hospitalization from COVID-19 compared to individuals with mild asthma or no asthma (83). Further research is needed to fully elucidate the complex relationship between asthma and COVID-19.

The limitations of our study primarily stem from its retrospective design. First, while data were collected, some variables were incomplete and had to be excluded from the analyses. Additionally, certain variables intended for inclusion could not be retrieved from hospital or testing platform records. Finally, due to local guidelines prioritizing testing for respiratory viruses such as SARS-CoV-2 and influenza, the number of tests conducted for other respiratory viruses was significantly lower. This limitation restricted the scope of analyses for these subgroups. Furthermore, our study was unable to precisely differentiate between the effects of influenza and COVID-19 vaccines on SARI risk, as the vaccination history recorded in our platform did not specify the type of vaccine received. Although the registry primarily captured COVID-19 vaccination data, influenza vaccination campaigns were reinforced during the study period, preventing a stratified analysis to assess the independent contribution of each vaccine type. Additionally, our dataset did not include exact vaccination dates, limiting our ability to evaluate the potential waning of immunity over time. Similarly, we lacked complete data on the timing of prior infections, whether caused by SARS-CoV-2 or other respiratory viruses, which restricts our capacity to fully explore the interplay between previous infections, vaccination, and SARI risk.

To ensure accurate and reliable results, additional studies should be conducted prospectively in diverse regions worldwide, either at the hospital or population level. Such studies would provide opportunities to include larger datasets and better control for confounding variables. Importantly, the findings of this study are limited to a single institution, which may constrain the generalizability of the results. Furthermore, more targeted testing strategies should be developed to explore associations with greater effect sizes, particularly in relation to respiratory virus infections other than influenza and SARS-CoV-2. Expanding on these findings requires further research within the same population and region, especially in this post-pandemic period, through prospective multicentric cohort studies. Additionally, future studies should focus on the genetic changes in RSV and influenza to assess their adaptation to reduced host exposure during the pandemic. Such research could provide valuable insights into viral evolution, inform public health strategies, and guide the development of vaccines and treatments.

In conclusion, the COVID-19 pandemic induced profound disruptions in the circulation patterns of respiratory viruses, significantly altering seasonal trends and reducing overall viral positivity rates. While post-pandemic recovery has been observed, influenza and other respiratory viruses have not fully reverted to their pre-pandemic seasonal dynamics, suggesting lasting epidemiological shifts. This study provides evidence that SARI remains strongly associated with key demographic factors and comorbidities, including advanced age, male sex, cardiovascular disease, obesity, and immunosuppression. Importantly, vaccination was consistently identified as a protective factor against SARI, reinforcing its crucial role in respiratory infection mitigation strategies. The dynamic interplay between viral competition, public health interventions, and human behavior likely contributed to the observed changes in SARI trends, emphasizing the need for ongoing surveillance and adaptive public health responses. As respiratory virus epidemiology continues to evolve in the post-pandemic era, it is imperative to sustain high-resolution surveillance efforts and refine public health policies to anticipate and mitigate future outbreaks. Strengthening vaccination programs, ensuring equitable access to diagnostic testing, and developing predictive models for respiratory virus resurgence will be critical in reducing morbidity and mortality associated with these infections. Further longitudinal studies are required to assess the long-term implications of these epidemiological shifts and to guide data-driven decision-making in infectious disease control.

## Data Availability

The raw data supporting the conclusions of this article will be made available by the authors, without undue reservation.

## Glossary

ARDS: Acute respiratory distress syndrome
COPD: Chronic obstructive pulmonary disease
GOF: Goodness-of-Fit
HAdV: Human adenovirus
HBoV: Human bocavirus
HCoV-229E: Human coronavirus 229E
HCoV-HKU1: Human coronavirus HKU1
HCoV-OC43: Human coronavirus OC43
HCoV-NL63: Human coronavirus NL63
HEV/HRV: Human enterovirus/rhinovirus
hMPV: Human metapneumovirus
HPIV: Human parainfluenza virus
HPIV1: Human parainfluenza virus 1
HPIV2: Human parainfluenza virus 2
HPIV3: Human parainfluenza virus 3
HPIV4: Human parainfluenza virus 4
ILI: Influenza-like illness
INFAH3: Influenza A virus H3
INFAH1N1: Influenza A virus H1N1
INFBVIC: Influenza B Victoria lineage
INFBYAM: Influenza B Yamagata lineage
ORVs. Other respiratory viruses (distinct from SARS-CoV-2 and influenza virus)
RSV: Respiratory syncytial virus
RT–PCR: Real-time polymerase chain reaction
SARIs: Severe acute respiratory infections
SARS-CoV-2: Severe acute respiratory syndrome coronavirus 2
