# Supplementary material for: Trends of respiratory viruses and factors associated with severe acute respiratory infection in patients presenting at a university hospital: a 6-year retrospective study across the COVID-19 pandemic
Source: Front Public Health. 2025 Mar 28;13:1494463. doi: 10.3389/fpubh.2025.1494463 (PMC11986719; doi:10.3389/fpubh.2025.1494463)
Supplement: Supplementary file 1 [file Data_Sheet_1.docx]

Supplementary Material

**Supplementary Table S1. Results of the interrupted time series analysis comparing LOESS-adjusted positivity rates and raw positivity rates for respiratory viruses across pandemic periods.**

**Supplementary Table S2. Results of the interrupted time series analysis using LOESS-adjusted positivity rates for respiratory viruses across pandemic periods, stratified by age in the non-adult population.**

**Supplementary Table S3. Univariate analyses of factors associated with severe acute respiratory infection during the study period.**

**Supplementary Table S4. Logistic regression models: factors associated with severe acute respiratory infection during the pre-pandemic period.**

**Supplementary Table S5. Logistic regression models: factors associated with severe acute respiratory infection during the post-pandemic period.**

**Supplementary Table S1. Results of the interrupted time series analysis comparing LOESS-adjusted positivity rates and raw positivity rates for respiratory viruses across pandemic periods.**

| **LOESS-adjusted positivity rates** | | | | | | | | | | | | | | | | | | | | | | | | |
| --- | --- | --- | --- | --- | --- | --- | --- | --- | --- | --- | --- | --- | --- | --- | --- | --- | --- | --- | --- | --- | --- | --- | --- | --- |
| **Variable** | **Influenza** | | | **Respiratory syncytial virus** | | | **Human enterovirus/rhinovirus** | | | **Human parainfluenza virus 1** | | | **Human adenovirus** | | | **Human metapneumovirus** | | | **Human bocavirus** | | | **Human coronavirus** | | |
|  | β | SE | *p* value | β | SE | *p* value | β | SE | *p* value | β | SE | *p* value | β | SE | *p* value | β | SE | *p* value | β | SE | *p* value | β | SE | *p* value |
| Constant (β0) | 45.33 | 1.04 | <0.001 | -0.96 | 0.29 | <0.001 | -0.28 | 0.17 | 0.10 | 0.35 | 0.17 | 0.04 | -0.05 | 0.03 | 0.08 | -0.24 | 0.06 | <0.001 | 0.00 | 0.02 | 0.94 | -0.24 | 0.04 | <0.001 |
| Time (β1) | -0.60 | 0.04 | <0.001 | 0.20 | 0.01 | <0.001 | 0.16 | 0.01 | <0.001 | 0.05 | 0.01 | <0.001 | 0.00 | 0.00 | 0.00 | 0.04 | 0.00 | <0.001 | 0.00 | 0.00 | 0.88 | 0.02 | 0.00 | <0.001 |
| Level | | | | | | | | | | | | | | | | | | | | | | | | |
| 2020-2021 (β2) | -44.17 | 1.86 | <0.001 | 1.20 | 0.51 | 0.02 | 5.51 | 0.30 | <0.001 | -0.38 | 0.30 | 0.21 | 0.45 | 0.05 | <0.001 | 1.10 | 0.11 | <0.001 | -0.05 | 0.03 | 0.11 | 1.43 | 0.06 | <0.001 |
| 2022 (β2) | -51.76 | 2.44 | <0.001 | -0.08 | 0.67 | 0.90 | 0.87 | 0.40 | 0.03 | -0.67 | 0.40 | 0.09 | -0.25 | 0.07 | <0.001 | 0.34 | 0.15 | 0.02 | 0.00 | 0.04 | 0.97 | 0.23 | 0.08 | 0.01 |
| Trend | | | | | | | | | | | | | | | | | | | | | | | | |
| 2020-2021 (β3) | -0.01 | 0.02 | 0.57 | 0.00 | 0.00 | 0.84 | -0.05 | 0.00 | <0.001 | 0.00 | 0.00 | 0.83 | 0.00 | 0.00 | <0.001 | -0.01 | 0.00 | <0.001 | 0.00 | 0.00 | <0.001 | -0.01 | 0.00 | <0.001 |
| 2022 (β3) | 0.11 | 0.01 | <0.001 | 0.01 | 0.00 | 0.03 | 0.00 | 0.00 | 0.45 | 0.00 | 0.00 | 0.25 | 0.00 | 0.00 | <0.001 | 0.00 | 0.00 | 0.65 | 0.00 | 0.00 | 1.00 | 0.00 | 0.00 | 0.41 |
|  | | | | | | | | | | | | | | | | | | | | | | | | |
| **Raw positivity rates** | | | | | | | | | | | | | | | | | | | | | | | | |
| **Variable** | **Influenza** | | | **Respiratory syncytial virus** | | | **Human enterovirus/rhinovirus** | | | **Human parainfluenza virus 1** | | | **Human adenovirus** | | | **Human metapneumovirus** | | | **Human bocavirus*** | | | **Human coronavirus** | | |
|  | β | SE | *p* value | β | SE | *p* value | β | SE | *p* value | β | SE | *p* value | β | SE | *p* value | β | SE | *p* value | β | SE | *p* value | β | SE | *p* value |
| Constant (β0) | 2.28 | 1.69 | 0.179 | -0.10 | 0.09 | 0.285 | -0.05 | 0.10 | 0.632 | 0.06 | 0.05 | 0.233 | 0.00 | 0.05 | 0.948 | -0.02 | 0.04 | 0.688 | - | - | - | -0.03 | 0.05 | 0.485 |
| Time (β1) | -0.02 | 0.03 | 0.564 | 0.01 | 0.00 | <0.001 | 0.01 | 0.00 | 0.003 | 0.00 | 0.00 | 0.397 | 0.00 | 0.00 | 0.576 | 0.00 | 0.00 | 0.039 | - | - | - | 0.00 | 0.00 | 0.206 |
| Level | | | | | | | | | | | | | | | | | | | | | | | | |
| 2020-2021 (β2) | -3.63 | 5.55 | 0.513 | -0.85 | 0.29 | 0.004 | 1.00 | 0.34 | 0.004 | -0.14 | 0.16 | 0.380 | 0.29 | 0.15 | 0.053 | -0.24 | 0.13 | 0.073 | - | - | - | 0.55 | 0.16 | <0.001 |
| 2022 (β2) | 14.81 | 6.93 | 0.033 | 0.05 | 0.36 | 0.893 | 0.64 | 0.43 | 0.136 | 0.09 | 0.20 | 0.649 | 0.02 | 0.19 | 0.918 | -0.14 | 0.17 | 0.411 | - | - | - | 0.14 | 0.19 | 0.460 |
| Trend | | | | | | | | | | | | | | | | | | | | | | | | |
| 2020-2021 (β3) | 0.01 | 0.03 | 0.772 | 0.01 | 0.00 | <0.001 | 0.00 | 0.00 | 0.061 | 0.00 | 0.00 | 0.443 | 0.00 | 0.00 | 0.117 | 0.00 | 0.00 | 0.022 | - | - | - | 0.00 | 0.00 | 0.006 |
| 2022 (β3) | -0.03 | 0.03 | 0.260 | 0.00 | 0.00 | 0.771 | 0.00 | 0.00 | 0.316 | 0.00 | 0.00 | 0.592 | 0.00 | 0.00 | 0.703 | 0.00 | 0.00 | 0.213 | - | - | - | 0.00 | 0.00 | 0.659 |

*For HBoV, the ITS analysis could not be performed using raw positivity rates due to insufficient data points

**Supplementary Table S2. Results of the interrupted time series analysis using LOESS-adjusted positivity rates for respiratory viruses across pandemic periods, stratified by age in the non-adult population.**

| **Age group < 5 years** | | | | | | | | | | | | | | | | | | |
| --- | --- | --- | --- | --- | --- | --- | --- | --- | --- | --- | --- | --- | --- | --- | --- | --- | --- | --- |
| **Variable** | **SARS-CoV-2** | | | **Influenza** | | | **Human enterovirus/rhinovirus** | | | **Respiratory syncytial virus** | | | **Human parainfluenza virus** | | | **Other respiratory viruses as a group*** | | |
|  | β | SE | *p* value | β | SE | *p* value | β | SE | *p* value | β | SE | *p* value | β | SE | *p* value | β | SE | *p* value |
| Constant (β0) | 0.00 | 11.10 | 1.00 | 28.82 | 9.38 | 0.00 | -1.08 | 2.15 | 0.62 | 0.00 | 2.97 | 1.00 | 0.00 | 1.55 | 1.00 | -1.08 | 5.28 | 0.84 |
| Time (β1) | 0.00 | 0.47 | 1.00 | -0.72 | 0.40 | 0.07 | 0.19 | 0.09 | 0.04 | 0.00 | 0.13 | 1.00 | 0.00 | 0.07 | 1.00 | 0.19 | 0.23 | 0.40 |
| Level | | | | | | | | | | | | | | | | | | |
| 2020-2021 (β2) | 10.41 | 13.46 | 0.44 | -23.23 | 11.37 | 0.04 | 1.08 | 2.61 | 0.68 | -4.83 | 3.60 | 0.18 | -0.05 | 1.88 | 0.98 | 1.73 | 6.40 | 0.79 |
| 2022 (β2) | 18.01 | 12.07 | 0.14 | -24.18 | 10.19 | 0.02 | 2.26 | 2.34 | 0.34 | -0.53 | 3.23 | 0.87 | 0.00 | 1.68 | 1.00 | 1.25 | 5.74 | 0.83 |
| Trend | | | | | | | | | | | | | | | | | | |
| 2020-2021 (β3) | 0.26 | 0.20 | 0.20 | -0.13 | 0.17 | 0.44 | 0.00 | 0.04 | 1.00 | 0.26 | 0.05 | 0.00 | 0.03 | 0.03 | 0.31 | 0.19 | 0.10 | 0.06 |
| 2022 (β3) | -0.21 | 0.11 | 0.06 | 0.11 | 0.09 | 0.27 | -0.01 | 0.02 | 0.61 | 0.03 | 0.03 | 0.33 | 0.00 | 0.02 | 1.00 | 0.06 | 0.05 | 0.30 |
|  | | | | | | | | | | | | | | | | | | |
| **Age group < 18 years** | | | | | | | | | | | | | | | | | | |
| **Variable** | **SARS-CoV-2** | | | **Influenza** | | | **Human enterovirus/rhinovirus** | | | **Respiratory syncytial virus** | | | **Human parainfluenza virus** | | | **Other respiratory viruses as a group*** | | |
|  | β | SE | *p* value | β | SE | *p* value | β | SE | *p* value | β | SE | *p* value | β | SE | *p* value | β | SE | *p* value |
| Constant (β0) | 0.00 | 7.25 | 1.00 | 8.55 | 7.16 | 0.23 | -3.24 | 2.88 | 0.26 | -4.33 | 2.40 | 0.07 | -0.56 | 0.78 | 0.48 | -5.47 | 3.52 | 0.12 |
| Time (β1) | 0.00 | 0.18 | 1.00 | 0.28 | 0.18 | 0.12 | 0.28 | 0.07 | 0.00 | 0.26 | 0.06 | 0.00 | 0.05 | 0.02 | 0.02 | 0.49 | 0.09 | <0.01 |
| Level | | | | | | | | | | | | | | | | | | |
| 2020-2021 (β2) | 18.20 | 8.96 | 0.04 | -7.04 | 8.84 | 0.43 | 2.39 | 3.55 | 0.50 | 1.03 | 2.97 | 0.73 | 0.49 | 0.97 | 0.62 | 2.69 | 4.36 | 0.54 |
| 2022 (β2) | 22.78 | 8.27 | 0.01 | 0.28 | 8.17 | 0.97 | 5.56 | 3.28 | 0.09 | 4.26 | 2.74 | 0.12 | 0.56 | 0.90 | 0.53 | 7.62 | 4.02 | 0.06 |
| Trend | | | | | | | | | | | | | | | | | | |
| 2020-2021 (β3) | 0.09 | 0.11 | 0.44 | -0.03 | 0.11 | 0.80 | 0.06 | 0.04 | 0.16 | 0.13 | 0.04 | 0.00 | 0.01 | 0.01 | 0.63 | 0.18 | 0.05 | <0.001 |
| 2022 (β3) | -0.29 | 0.09 | 0.00 | 0.05 | 0.09 | 0.56 | -0.03 | 0.04 | 0.37 | 0.01 | 0.03 | 0.75 | 0.00 | 0.01 | 1.00 | -0.01 | 0.05 | 0.83 |

***Other respiratory viruses as a group: Includes all respiratory viruses other than influenza and SARS-CoV-2.**

**Supplementary Table S3. Univariate analyses of factors associated with severe acute respiratory infection during the study period.**

| **Prepandemic period** | | | | | | **Postpandemic period** | | | | | |
| --- | --- | --- | --- | --- | --- | --- | --- | --- | --- | --- | --- |
| **General population with positive tests** | | | | | | **General population with positive tests 0-18 years** | | | | | |
| **Variable** | **Obs* (n)**   \| **SARI**  **Absent** \| **SARI**  **Present** \| \| --- \| --- \| | **Total**  **(n)** | **SARI Absent n,(%)** | **SARI Present n,(%)** | ***p* value** | **Variable** | **Obs* (n)**   \| **SARI**  **Absent** \| **SARI**  **Present** \| \| --- \| --- \| | **Total**  **(n)** | **SARI Absent n,(%)** | **SARI Present n,(%)** | ***p* value** |
| Male sex | 76 \| 122 | 95 | 23 (30.3) | 72 (59.0) | <0.001 | Male sex | 336 \| 124 | 241 | 176 (52.4) | 65 (52.4) | 1.000 |
| Comorbilities | 71 \| 60 | 88 | 28 (39.4) | 60 (100.0) | <0.001 | Comorbilities | 336 \| 46 | 98 | 55 (16.4) | 43 (93.5) | <0.001 |
| Diabetes | 71 \| 60 | 18 | 2 (2.8) | 16 (26.7) | <0.001 | Diabetes | 334 \| 46 | 9 | 6 (1.8) | 3 (6.5) | 0.083 |
| Cardiovascular disease | 71 \| 60 | 8 | 1 (1.4) | 7 (11.7) | 0.023 | Cardiovascular disease | 334 \| 46 | 7 | 4 (1.2) | 3 (6.5) | 1.000 |
| Obesity | 71 \| 64 | 32 | 8 (11.3) | 24 (37.5) | <0.001 | Obesity | 334 \| 46 | 6 | 5 (1.5) | 1 (2.2) | 0.541 |
| Asthma | 71 \| 60 | 24 | 8 (11.3) | 16 (26.7) | 0.041 | Asthma | 334 \| 46 | 16 | 11 (3.3) | 5 (10.9) | 0.045 |
| COPD | 72 \| 93 | 10 | 1 (1.4) | 9 (9.7) | 0.044 | COPD | - | - | - | - | - |
| Smoking | 71 \| 60 | 7 | 1 (1.4) | 6 (10.0) | 0.047 | Smoking | 334 \| 46 | 6 | 6 (1.8) | 0 (0.0) | 1.000 |
| Hypertension | 71 \| 60 | 19 | 1 (1.4) | 18 (30.0) | <0.001 | Hypertension | 334 \| 46 | 6 | 5 (1.5) | 1 (2.2) | 0.541 |
| Immunosuppression status | 71 \| 60 | 9 | 2 (2.8) | 7 (11.7) | 0.079 | Immunosuppression status | 334 \| 46 | 21 | 8 (2.4) | 13 (28.3) | <0.001 |
| PLHIV | 71 \| 60 | 2 | 0 (0.0) | 2 (3.3) | 0.208 | PLHIV | 334 \| 46 | 1 | 1 (0.3) | 0 (0.0) | 1.000 |
| Chronic kidney disease | 71 \| 60 | 1 | 0 (0.0) | 1 (1.7) | 0.458 | Chronic kidney disease | 334 \| 46 | 4 | 4 (1.2) | 0 (0.0) | 1.000 |
| - | - | - | - | - | - | Vaccination | 334 \| 46 | 41 | 38 (38.8) | 3 (6.5) | <0.001 |
| Days from symptom onset to testing | 76 \| 122 | 2.0  (1.0-4.0) | 2.0  (1.0-3.0) | 3.0  (2.0-5.0) | <0.001 | Days from symptom onset to testing | 336 \| 124 | 2.0  (1.0-3.0) | 2.0  (1.0-3.0) | 2.0  (1.0-4.0) | <0.001 |
| **Influenza** | | | | | | **General population with positive tests 18-65 years** | | | | | |
| **Variable** | **Obs* (n)**   \| **SARI**  **Absent** \| **SARI**  **Present** \| \| --- \| --- \| | **Total**  **(n)** | **SARI Absent n,(%)** | **SARI Present n,(%)** | ***p* value** | **Variable** | **Obs* (n)**   \| **SARI**  **Absent** \| **SARI**  **Present** \| \| --- \| --- \| | **Total**  **(n)** | **SARI Absent n,(%)** | **SARI Present n,(%)** | ***p* value** |
| Male sex | 73 \| 70 | 92 | 52 (71.2) | 40 (57.1) | 0.001 | Male sex | 5596 \| 811 | 2627 | 2176 (38.9) | 451 (55.6) | <0.001 |
| Comorbilities | 69 \| 41 | 68 | 27 (39.1) | 41 (100) | <0.001 | Comorbilities | 5582 \| 495 | 2029 | 1600 (28.7) | 429 (86.7) | <0.001 |
| Diabetes | 69 \| 41 | 12 | 2 (2.9) | 10 (24.4) | <0.001 | Diabetes | 4988 \| 495 | 580 | 413 (8.3) | 167 (33.7) | <0.001 |
| Cardiovascular disease | 69 \| 41 | 6 | 1 (1.4) | 5 (12.2) | 0.026 | Cardiovascular disease | 4988 \| 495 | 53 | 26 (0.5) | 27 (5.5) | <0.001 |
| Obesity | 69 \| 45 | 28 | 8 (11.6) | 20 (44.4) | <0.001 | Obesity | 4988 \| 495 | 518 | 393 (7.9) | 125 (25.3) | <0.001 |
| Asthma | 69 \| 41 | 19 | 8 (11.6) | 11 (26.8) | 0.075 | Asthma | 4988 \| 495 | 242 | 211 (4.2) | 31 (6.3) | 0.047 |
| COPD | 69 \| 41 | 7 | 1 (1.4) | 6 (14.6) | 0.010 | COPD | 4988 \| 495 | 29 | 9 (0.2) | 20 (4.0) | <0.001 |
| Smoking | 69 \| 41 | 7 | 1 (1.4) | 6 (14.6) | 0.010 | Smoking | 4988 \| 495 | 321 | 252 (5.1) | 69 (13.9) | <0.001 |
| Hypertension | 69 \| 41 | 14 | 1 (1.4) | 13 (31.7) | <0.001 | Hypertension | 4988 \| 495 | 630 | 464 (9.3) | 166 (33.5) | <0.001 |
| Immunosuppression status | 69 \| 41 | 5 | 2 (2.9) | 3 (7.3) | 0.359 | Immunosuppression status | 4988 \| 495 | 169 | 119 (2.4) | 50 (10.1) | <0.001 |
| PLHIV | 69 \| 41 | 1 | 0 (0.0) | 1 (2.4) | 0.373 | PLHIV | 4988 \| 495 | 53 | 40 (0.8) | 13 (2.6) | <0.001 |
| Chronic kidney disease | - | - | - | - | - | Chronic kidney disease | 4988 \| 495 | 101 | 67 (1.3) | 34 (6.9) | <0.001 |
| Age | 73 \| 70 | 25.0  (11.0-46.0) | 21.0  (8.0-30.0) | 43.0  (15.0-61.0) | <0.001 | Vaccination | 3240 \| 495 | 2270 | 2167 (66.9) | 103 (20.8) | <0.001 |
| Days from symptom onset to testing | 73 \| 70 | 2.0  (1.0-4.0) | 2.0  (1.0-3.0) | 3.0  (2.0-5.0) | <0.001 | Days from symptom onset to testing | 5596 \| 811 | 2.0  (1.0-3.0) | 2.0  (1.0-3.0) | 3.0  (2.0-5.0) | <0.001 |
| Ct value of RT-PCR | 73 \| 70 | 32.0  (27.0-35.0) | 34.0  (30.0-36.0) | 30.0  (25.2-34.0) | 0.002 |  | | | | | |
| **Other respiratory viruses** | | | | | | **General population with positive tests >65 years** | | | | | |
| **Variable** | **Obs* (n)**   \| **SARI**  **Absent** \| **SARI**  **Present** \| \| --- \| --- \| | **Total**  **(n)** | **SARI Absent n,(%)** | **SARI Present n,(%)** | ***p* value** | **Variable** | **Obs* (n)**   \| **SARI**  **Absent** \| **SARI**  **Present** \| \| --- \| --- \| | **Total**  **(n)** | **SARI Absent n,(%)** | **SARI Present n,(%)** | ***p* value** |
| Male sex | 3 \| 54 | 35 | 2 (66.7) | 33 (61.1) | 1.000 | Male sex | 380 \| 369 | 374 | 181 (47.6) | 193 (52.3) | 0.228 |
| Comorbilities | 2 \| 20 | 21 | 1 (50.0) | 20 (100.0) | 0.091 | Comorbilities | 380 \| 279 | 514 | 249 (65.5) | 265 (95.0) | <0.001 |
| Diabetes | 2 \| 20 | 6 | 0 (0.0) | 6 (30.0) | 1.000 | Diabetes | 359 \| 279 | 251 | 117 (32.6) | 134 (48.0) | <0.001 |
| Cardiovascular disease | 2 \| 20 | 2 | 0 (0.0) | 2 (10.0) | 1.000 | Cardiovascular disease | 359 \| 279 | 48 | 13 (3.6) | 35 (12.5) | <0.001 |
| Obesity | 2 \| 20 | 4 | 0 (0.0) | 4 (20.0) | 1.000 | Obesity | 359 \| 279 | 77 | 22 (6.1) | 55 (19.7) | <0.001 |
| Asthma | 2 \| 20 | 5 | 0 (0.0) | 5 (25.0) | 1.000 | Asthma | 359 \| 279 | 20 | 8 (2.2) | 12 (4.3) | 0.207 |
| COPD | 3 \| 53 | 3 | 0 (0.0) | 3 (5.7) | 1.000 | COPD | 359 \| 279 | 63 | 22 (6.1) | 41 (14.7) | <0.001 |
| Smoking | - | - | - | - | - | Smoking | 359 \| 279 | 58 | 20 (5.6) | 38 (13.6) | <0.001 |
| Hypertension | 2 \| 20 | 5 | 0 (0.0) | 5 (25.0) | 1.000 | Hypertension | 359 \| 279 | 344 | 167 (46.5) | 177 (63.4) | <0.001 |
| Immunosuppression status | 2 \| 20 | 5 | 0 (0.0) | 5 (25.0) | 1.000 | Immunosuppression status | 359 \| 279 | 36 | 11 (3.1) | 25 (9.0) | 0.002 |
| PLHIV | 2 \| 20 | 1 | 0 (0.0) | 1 (5.0) | 1.000 | PLHIV | 359 \| 279 | 3 | 0 (0.0) | 3 (1.1) | 0.083 |
| Chronic kidney disease | 2 \| 20 | 1 | 0 (0.0) | 1 (5.0) | 1.000 | Chronic kidney disease | 359 \| 279 | 26 | 10 (2.8) | 16 (5.7) | 0.095 |
| Age | 3 \| 54 | 3.0 (1.0-16.0) | 22.0  (12.5-26.5) | 3.0 (1.0-12.5) | 0.269 | Vaccination | 275 \| 279 | 122 | 80 (29.1) | 42 (15.1) | <0.001 |
| Days from symptom onset to testing | 3 \| 54 | 3.0  (2.0-5.0) | 2.0 (2.0-2.5) | 3.0  (2.0-5.0) | 0.386 | Days from symptom onset to testing | 380 \| 369 | 3.0 (1.0-4.0) | 2.0 (1.0-4.0) | 3.0 (2.0-5.0) | <0.001 |
|  |  |  |  |  |  | **SARS-CoV2** | | | | | |
|  | | | | | | **Variable** | **Obs* (n)**   \| **SARI**  **Absent** \| **SARI**  **Present** \| \| --- \| --- \| | **Total**  **(n)** | **SARI Absent n,(%)** | **SARI Present n,(%)** | ***p* value** |
|  |  |  |  |  |  | Male sex | 5448 \| 1057 | 2767 | 2178 (40.0) | 589 (55.7) | <0.001 |
|  |  |  |  |  |  | Comorbilities | 5438 \| 645 | 2259 | 1661 (30.5) | 598 (92.7) | <0.001 |
|  |  |  |  |  |  | Diabetes | 4842 \| 645 | 752 | 491 (10.1) | 261 (40.5) | <0.001 |
|  |  |  |  |  |  | Cardiovascular disease | 4842 \| 645 | 85 | 35 (0.7) | 50 (7.8) | <0.001 |
|  |  |  |  |  |  | Obesity | 4842 \| 645 | 530 | 369 (7.6) | 161 (25.0) | <0.001 |
|  |  |  |  |  |  | Asthma | 4842 \| 645 | 208 | 185 (3.8) | 23 (3.6) | 0.835 |
|  |  |  |  |  |  | COPD | 4842 \| 645 | 61 | 26 (0.5) | 35 (5.4) | <0.001 |
|  |  |  |  |  |  | Smoking | 4842 \| 645 | 320 | 239 (4.9) | 81 (12.6) | <0.001 |
|  |  |  |  |  |  | Hypertension | 4842 \| 645 | 882 | 587 (12.1) | 295 (45.7) | <0.001 |
|  |  |  |  |  |  | Immunosuppression status | 4842 \| 645 | 177 | 107 (2.2) | 70 (10.9) | <0.001 |
|  |  |  |  |  |  | PLHIV | 4842 \| 645 | 45 | 32 (0.7) | 13 (2.0) | <0.001 |
|  |  |  |  |  |  | Chronic kidney disease | 4842 \| 645 | 103 | 66 (1.4) | 37 (5.7) | <0.001 |
|  |  |  |  |  |  | Vaccination | 2947 \| 645 | 1822 | 1733 (58.8%) | 89 (13.8%) | <0.001 |
|  |  |  |  |  |  | Coinfections between SARS-CoV-2 and Influenza | 5448 \| 1057 | 91 | 85 (4.4%) | 6 (1.9%) | 0.047 |
|  |  |  |  |  |  | Age | 5448 \| 1057 | 40.0  (29.0-53.0) | 38.0  (28.0-51.0) | 55.0  (39.0-68.0) | <0.001 |
|  |  |  |  |  |  | Days from symptom onset to testing | 5448 \| 1057 | 2.0  (1.0-3.0) | 2.0  (1.0-3.0) | 3.0  (2.0-5.0) | <0.001 |
|  |  |  |  |  |  | RT-PCR Ct value | 5448 \| 1057 | 25.0  (21.0-32.0) | 25.0  (21.0-33.0) | 27.0  (22.0-32.0) | <0.001 |
|  |  |  |  |  |  | **Influenza** | | | | | |
|  | | | | | | **Variable** | **Obs* (n)**   \| **SARI**  **Absent** \| **SARI**  **Present** \| \| --- \| --- \| | **Total**  **(n)** | **SARI Absent n,(%)** | **SARI Present n,(%)** | ***p* value** |
|  |  |  |  |  |  | Male sex | 868 \| 168 | 421 | 350 (40.3) | 71 (42.3) | 0.702 |
|  |  |  |  |  |  | Comorbilities | 866 \| 126 | 338 | 247 (28.5) | 91 (72.2) | <0.001 |
|  |  |  |  |  |  | Diabetes | 844 \| 126 | 82 | 49 (5.8) | 33 (26.2) | <0.001 |
|  |  |  |  |  |  | Cardiovascular disease | 844 \| 126 | 18 | 8 (0.9) | 10 (7.9) | <0.001 |
|  |  |  |  |  |  | Obesity | 844 \| 126 | 62 | 49 (5.8) | 13 (10.3) | 0.083 |
|  |  |  |  |  |  | Asthma | 844 \| 126 | 60 | 43 (5.1) | 17 (13.5) | <0.001 |
|  |  |  |  |  |  | COPD | 844 \| 126 | 23 | 6 (0.7) | 17 (13.5) | <0.001 |
|  |  |  |  |  |  | Smoking | 844 \| 126 | 56 | 39 (4.6) | 17 (13.5) | <0.001 |
|  |  |  |  |  |  | Hypertension | 844 \| 126 | 88 | 53 (6.3) | 35 (27.8) | <0.001 |
|  |  |  |  |  |  | Immunosuppression status | 844 \| 126 | 37 | 26 (3.1) | 11 (8.7) | 0.005 |
|  |  |  |  |  |  | PLHIV | 844 \| 126 | 9 | 8 (0.9) | 1 (0.8) | 1.000 |
|  |  |  |  |  |  | Chronic kidney disease | 844 \| 126 | 20 | 15 (1.8) | 5 (4.0) | 0.201 |
|  |  |  |  |  |  | Vaccination | 707 \| 126 | 663 | 605 (85.6) | 58 (46.0) | <0.001 |
|  |  |  |  |  |  | Coinfections between SARS-CoV-2 and Influenza | 868 \| 168 | 91 | 85 (9.8) | 6 (3.6) | 0.014 |
|  |  |  |  |  |  | Age | 868 \| 168 | 30.0  (23.0-43.0) | 29.0  (23.0-40.0) | 37.0  (20.8-62.3) | <0.001 |
|  |  |  |  |  |  | Days from symptom onset to testing | 868 \| 168 | 2.0  (1.0-2.0) | 2.0  (1.0-2.0) | 2.0  (1.8-3.0) | <0.001 |
|  |  |  |  |  |  | RT-PCR Ct value | 868 \| 168 | 26.0  (22.0-29.0) | 26.0  (22.0-29.0) | 26.0  (23.0-30.0) | 0.113 |
|  |  |  |  |  |  | **Other respiratory viruses** | | | | | |
|  | | | | | | **Variable** | **Obs* (n)**   \| **SARI**  **Absent** \| **SARI**  **Present** \| \| --- \| --- \| | **Total**  **(n)** | **SARI Absent n,(%)** | **SARI Present n,(%)** | ***p* value** |
|  |  |  |  |  |  | Male sex | 33 \| 84 | 61 | 14 (42.4) | 47 (56.0) | 0.266 |
|  |  |  |  |  |  | Comorbilities | 31 \| 54 | 65 | 13 (41.9) | 52 (96.3) | <0.001 |
|  |  |  |  |  |  | Diabetes | 30 \| 54 | 14 | 1 (3.3) | 13 (24.1) | 0.015 |
|  |  |  |  |  |  | Cardiovascular disease | 30 \| 54 | 4 | 0 (0.0) | 4 (7.4) | 0.292 |
|  |  |  |  |  |  | Obesity | 30 \| 54 | 11 | 2 (6.7) | 9 (16.7) | 0.313 |
|  |  |  |  |  |  | Asthma | 30 \| 54 | 13 | 4 (13.3) | 9 (16.7) | 0.763 |
|  |  |  |  |  |  | COPD | 30 \| 54 | 10 | 0 (0.0) | 10 (18.5) | 0.012 |
|  |  |  |  |  |  | Smoking | 30 \| 54 | 13 | 2 (6.7) | 11 (20.4) | 0.123 |
|  |  |  |  |  |  | Hypertension | 30 \| 54 | 17 | 0 (0.0) | 17 (31.5) | <0.001 |
|  |  |  |  |  |  | Immunosuppression status | 30 \| 54 | 11 | 6 (20.0) | 5 (9.3) | 0.289 |
|  |  |  |  |  |  | PLHIV | 30 \| 54 | 4 | 2 (6.7) | 2 (3.7) | 0.614 |
|  |  |  |  |  |  | Chronic kidney disease | 30 \| 54 | 8 | 0 (0.0) | 8 (14.8) | 0.046 |
|  |  |  |  |  |  | Vaccination | 19 \| 54 | 11 | 8 (42.1) | 3 (5.6) | <0.001 |
|  |  |  |  |  |  | Age | 33 \| 84 | 28.5  (2.0-61.0) | 30.0  (20.0-55.0) | 22.5  (1.0-65.5) | 0.339 |
|  |  |  |  |  |  | Days from symptom onset to testing | 33 \| 84 | 3.0  (2.0-4.0) | 2.0  (1.0-4.0) | 3.0  (2.0-4.0) | 0.009 |

COPD: Chronic Obstructive Pulmonary Disease; PLHIV: Person diagnosed with Human Immunodeficiency Virus; SARI Absent: (Cases without severe acute respiratory infection; SARI Present: Cases with severe acute respiratory infection;

*Observations frequency.

**Supplementary Table S4. Logistic regression models: factors associated with severe acute respiratory infection during the pre-pandemic period.**

| **General population** | | | | | | |
| --- | --- | --- | --- | --- | --- | --- |
| **term** | **OR adj** | **std.error** | **statistic** | **conf.low** | **conf.high** | ***p* value** |
| Age | 1.040 | 0.010 | 3.840 | 1.020 | 1.060 | <0.001 |
| Male sex | 3.180 | 0.485 | 2.390 | 1.250 | 8.510 | 0.017 |
| Days from symptom onset to testing | 1.320 | 0.116 | 2.380 | 1.070 | 1.690 | 0.017 |
| Obesity | 2.570 | 0.588 | 1.600 | 0.818 | 8.430 | 0.109 |
| Asthma | 3.140 | 0.577 | 1.990 | 1.020 | 10.000 | 0.047 |
| McFadden Pseudo R2: 0.345. Hosmer and Lemeshow goodness of fit (GOF) test. X-squared = 5.7423, df = 3, p-value = 0.1248 | | | | | | |
| **Influenza** | | | | | | |
| **Term** | **OR adj** | **std.Error** | **statistic** | **conf.low** | **conf.high** | ***p* value** |
| Age | 1.05 | 0.017 | 2.78 | 1.02 | 1.09 | 0.005 |
| Male sex | 10.7 | 0.844 | 2.81 | 2.33 | 69.3 | 0.005 |
| Days from symptom onset to testing | 1.5 | 0.19 | 2.15 | 1.08 | 2.29 | 0.032 |
| RT-PCR Ct value | 0.786 | 0.086 | -2.79 | 0.649 | 0.916 | 0.005 |
| Asthma | 7.72 | 1.09 | 1.87 | 1.01 | 82 | 0.062 |
| Obesity | 13.7 | 1.06 | 2.47 | 2.03 | 147 | 0.014 |
| McFadden Pseudo R2: 0.611. Hosmer and Lemeshow goodness of fit (GOF) test. X-squared = 3.9648, df = 8, p-value = 0.8603 | | | | | | |
| **Other respiratory viruses** | | | | | | |
| **term** | **OR adj** | **std.error** | **statistic** | **conf.low** | **conf.high** | ***p* value** |
| Age | 0.994 | 0.0227 | -0.255 | 0.955 | 1.06 | 0.799 |
| Male sex | 0.626 | 1.29 | -0.363 | 0.027 | 7.35 | 0.716 |
| Days from symptom onset to testing | 1.38 | 0.337 | 0.951 | 0.795 | 3.15 | 0.342 |
| McFadden Pseudo R2: 0.05. Hosmer and Lemeshow goodness of fit (GOF) test. X-squared = 1.8711, df = 3, p-value = 0.5996 | | | | | | |

**Supplementary Table S5. Logistic regression models: factors associated with severe acute respiratory infection during the post-pandemic period.**

| **General population, age 0-18** | | | | | | | |
| --- | --- | --- | --- | --- | --- | --- | --- |
| **Variable** | **OR** | **std.error** | **statistic** | **conf.low** | **conf.high** | **p.value** | |
| Age | 0.30 | 0.53 | -2.31 | 0.10 | 0.81 | 0.02 | |
| Days from symptom onset to testing | 1.07 | 0.40 | 0.17 | 0.49 | 2.35 | 0.87 | |
| Immunosuppression status | 1.22 | 0.13 | 1.51 | 0.95 | 1.58 | 0.13 | |
| Vaccination | 3.80 | 0.53 | 2.53 | 1.38 | 11.20 | 0.01 | |
| Asthma | 0.172 | 0.660 | -2.670 | 0.038 | 0.554 | 0.008 | |
| McFadden Pseudo R2: 0.345. Hosmer and Lemeshow goodness of fit (GOF) test. X-squared = 5.7423, df = 3, p-value = 0.1248 | | | | | | | |
| **General population, age 18-65** | | | | | | | |
| **Variable** | **OR** | **std.error** | **statistic** | **conf.low** | **conf.high** | | **p.value** |
| Male sex | 1.5 | 0.109 | 3.73 | 1.21 | 1.86 | | <0.001 |
| Days of symptoms to test | 1.27 | 0.028 | 8.81 | 1.21 | 1.35 | | <0.001 |
| Vaccination | 0.291 | 0.139 | -8.89 | 0.221 | 0.381 | | <0.001 |
| Diabetes | 1.77 | 0.126 | 4.56 | 1.38 | 2.27 | | <0.001 |
| Cardiovascular disease | 3.26 | 0.319 | 3.7 | 1.74 | 6.11 | | <0.001 |
| Obesity | 1.38 | 0.132 | 2.43 | 1.06 | 1.78 | | 0.015 |
| Smoking | 1.09 | 0.163 | 0.506 | 0.785 | 1.49 | | 0.613 |
| Hypertension | 1.33 | 0.128 | 2.2 | 1.03 | 1.7 | | 0.028 |
| Immunosuppression status | 1.94 | 0.198 | 3.36 | 1.31 | 2.85 | | <0.001 |
| Chronic kidney disease | 1.73 | 0.246 | 2.22 | 1.06 | 2.78 | | 0.026 |
| McFadden Pseudo R2: 0.195. Hosmer and Lemeshow goodness of fit (GOF) test. X-squared = 1.0057, df = 3, p-value = 0.7999. | | | | | | | |
| **General population, age >65 years** | | | | | | | |
| **Variable** | **OR** | **std.error** | **statistic** | **conf.low** | **conf.high** | | **p.value** |
| Male sex | 0.913 | 0.182 | -0.501 | 0.638 | 1.3 | | 0.616 |
| Days from symptom onset to testing | 1.24 | 0.048 | 4.41 | 1.13 | 1.36 | | <0.001 |
| Vaccination | 0.594 | 0.229 | -2.27 | 0.377 | 0.927 | | 0.023 |
| Cardiovascular disease | 2.29 | 0.354 | 2.35 | 1.17 | 4.73 | | 0.019 |
| Obesity | 2.23 | 0.281 | 2.86 | 1.3 | 3.93 | | 0.004 |
| Immunosuppression status | 1.89 | 0.398 | 1.6 | 0.882 | 4.26 | | 0.111 |
| COPD | 1.99 | 0.292 | 2.35 | 1.13 | 3.57 | | 0.019 |
| McFadden Pseudo R2: 0.229. Hosmer and Lemeshow goodness of fit (GOF) test. X-squared = 3.1544, df = 3, p-value = 0.3684 | | | | | | | |
| **SARS-CoV-2 cases** | | | | | | | |
| **Variable** | **OR** | **std.error** | **statistic** | **conf.low** | **conf.high** | | **p.value** |
| Age | 1.03 | 0.003 | 8.91 | 1.02 | 1.03 | | <0.001 |
| Male sex | 1.39 | 0.101 | 3.25 | 1.14 | 1.69 | | 0.001 |
| Days of symptoms to test | 1.26 | 0.024 | 9.43 | 1.2 | 1.32 | | <0.001 |
| Vaccination | 0.259 | 0.135 | -10 | 0.199 | 0.337 | | <0.001 |
| Diabetes | 1.4 | 0.112 | 2.99 | 1.12 | 1.74 | | 0.003 |
| Cardiovascular disease | 2.88 | 0.257 | 4.11 | 1.75 | 4.79 | | <0.001 |
| COPD | 2.34 | 0.302 | 2.81 | 1.3 | 4.25 | | 0.005 |
| Asthma | 0.527 | 0.254 | -2.53 | 0.313 | 0.849 | | 0.012 |
| HIV Immunosuppression | 2.79 | 0.186 | 5.52 | 1.93 | 4 | | <0.001 |
| McFadden Pseudo R2: 0.242. Hosmer and Lemeshow goodness of fit (GOF) test. X-squared = 0.22467, df = 3, p-value = 0.9735 | | | | | | | |
| **Influenza cases** | | | | | | | |
| **Variable** | **OR** | **std.error** | **statistic** | **conf.low** | **conf.high** | | **p.value** |
| Age | 1.03 | 0.006 | 5.05 | 1.02 | 1.04 | | <0.001 |
| Male sex | 0.999 | 0.233 | -0.002 | 0.63 | 1.58 | | 0.998 |
| Days of symptoms to test | 1.48 | 0.107 | 3.65 | 1.2 | 1.83 | | <0.001 |
| SARS-CoV-2 and Influenza coinfection | 0.361 | 0.552 | -1.84 | 0.108 | 0.969 | | 0.065 |
| Diabetes | 1.86 | 0.313 | 1.98 | 0.996 | 3.41 | | 0.047 |
| Obesity | 0.966 | 0.4 | -0.086 | 0.426 | 2.06 | | 0.932 |
| Chronic kidney disease | 0.651 | 0.629 | -0.683 | 0.173 | 2.11 | | 0.494 |
| Smoking | 1.98 | 0.329 | 2.07 | 1.02 | 3.73 | | 0.038 |
| Asthma | 2.22 | 0.359 | 2.23 | 1.08 | 4.42 | | 0.026 |
| HIV Immunosuppression | 1.95 | 0.435 | 1.54 | 0.807 | 4.5 | | 0.124 |
| Vaccination | 0.244 | 0.247 | -5.7 | 0.15 | 0.397 | | <0.001 |
| McFadden Pseudo R2: 0.235. Hosmer and Lemeshow goodness of fit (GOF) test. X-squared = 7.7201, df = 8, p-value = 0.4613 | | | | | | | |
| **Other respiratory viruses cases** | | | | | | | |
| **Variable** | **OR** | **std.error** | **statistic** | **conf.low** | **conf.high** | | **p.value** |
| Age | 1.01 | 0.01 | 1.23 | 0.993 | 1.03 | | 0.218 |
| Male sex | 1.56 | 0.573 | 0.778 | 0.514 | 4.98 | | 0.437 |
| Smoking | 1.09 | 0.981 | 0.087 | 0.173 | 9.43 | | 0.931 |
| Asthma | 1.86 | 0.281 | 2.22 | 1.11 | 3.41 | | 0.027 |
| Days from symptom onset to testing | 1.65 | 0.192 | 2.62 | 1.17 | 2.5 | | 0.009 |
| McFadden Pseudo R2: 0.229. Hosmer and Lemeshow goodness of fit (GOF) test. X-squared = 4.7701, df = 3, p-value = 0.1894 | | | | | | | |
